# When phosphorus takes over: hyperconjugation-driven intramolecular O–H⋯O=P hydrogen bonds beyond classical RAHB

**DOI:** 10.1039/d6ra09698k

**Published:** 2026-11-10

**Authors:** Anna Pietrzak, Przemysław Nowak, Silvia C. Capelli, Marta K. Dudek, Wojciech M. Wolf

**Affiliations:** a Institute of General and Ecological Chemistry, Faculty of Chemistry, Łódź University of Technology Żeromskiego 114 90-543 Łódź Poland anna.pietrzak.1@p.lodz.pl; b Centre of Molecular and Macromolecular Studies, Polish Academy of Sciences Sienkiewicza 112 90-363 Łódź Poland; c BioMedChem Doctoral School of University of Lodz and Lodz Institutes of the Polish Academy of Sciences, University of Łódź Matejki 21/23 90-237 Łódź Poland; d ISIS Neutron and Muon Facility, Rutherford Appleton Laboratory Didcot OX11 0QX UK

## Abstract

Intramolecular hydrogen bonds embedded in conjugated frameworks are commonly rationalized within the resonance-assisted hydrogen bonding (RAHB) paradigm, in which π-electron delocalization cooperatively strengthens the O–H⋯O interaction. Here, we demonstrate that RAHB-like geometries can arise from a fundamentally different electronic mechanism. A family of phosphorylated hydroquinolinols featuring rare intramolecular O–H⋯O=P hydrogen bonds was investigated using single-crystal X-ray and neutron diffraction, NBO analysis, and solution-state NMR and IR spectroscopy. Although the hydrogen-bond-closed quasi-rings display structural signatures reminiscent of classical RAHB systems, bond-length equalization is limited and exhibits only minor variation across the investigated substitution series. Electronic structure analysis reveals that the phosphoryl group acts as a σ-resonance and negative-hyperconjugation-dominated hydrogen-bond acceptor, indicating that classical cooperative π-resonance is not the dominant factor governing hydrogen-bond strength within the investigated series. As a result, these O–H⋯O=P interactions are strong, persistent in solution, and exhibit only limited sensitivity to the structural modifications examined in the present series. These findings establish phosphoryl-centered intramolecular hydrogen bonds as a distinct electronic regime beyond classical RAHB and extend the concept of resonance assistance to heteronuclear, hyperconjugation-dominated frameworks.

## Abstract 2

Intramolecular O–H⋯O=P hydrogen bonds display RAHB-like geometries but arise from a phosphorus-driven electronic origin.

## Graphical abstract

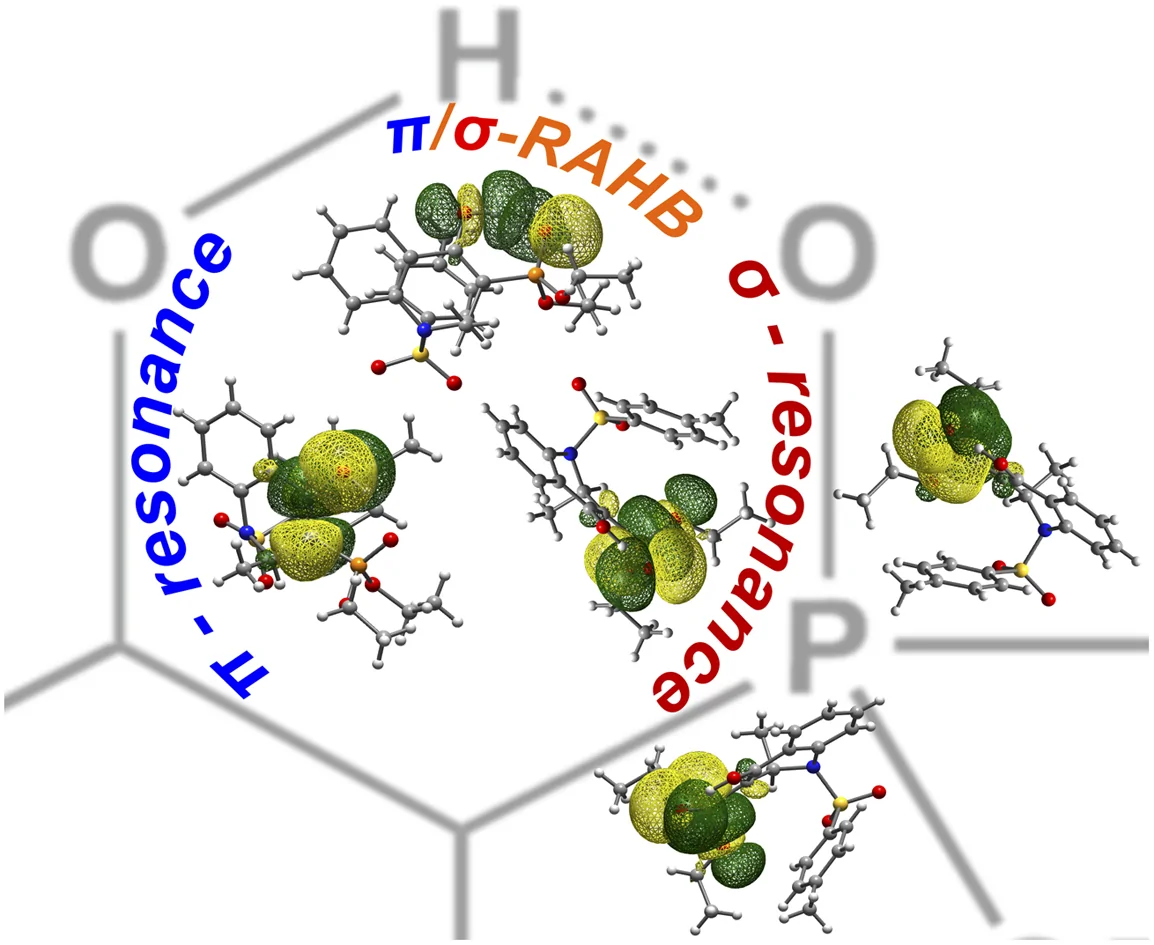

## Introduction

Intramolecular hydrogen bonds (intraHBs) embedded in conjugated frameworks are typically rationalized within Gilli's resonance-assisted hydrogen bonding (RAHB)^1^ paradigm, where π-electron delocalization cooperatively enhances hydrogen-bond strength and leads to characteristic bond-length equalization within the hydrogen-bonded quasi-ring, resulting in an “aromatic-like” delocalization signature.^2,3^ In this framework, the strength of the hydrogen bond is intrinsically linked to the extent of π-conjugation, and substituent effects are transmitted through the π-system.^4,5^

Despite the success of this model, it remains an open question whether strong intramolecular hydrogen bonds necessarily require π-conjugated frameworks, or whether alternative electronic mechanisms can sustain comparable interactions. Alternative concepts such as the Mills–Nixon effect^6,7^ and resonance-inhibited hydrogen bonds (RIHB)^8–11^ demonstrate that not every “resonance-accompanied hydrogen bond” can be straightforwardly classified as RAHB. Current understanding emphasizes that the atom types, resonance topology, and planarity jointly determine whether resonance cooperates with or counteracts hydrogen-bond formation.^9,10^ In this context, systems containing highly polarized heteronuclear acceptors, such as phosphoryl groups, offer the possibility of hydrogen-bond stabilization dominated by σ-type delocalization (hyperconjugation) rather than classical π-resonance. To date, however, most RAHB and RIHB studies focus on O, N, or S donors and acceptors connected *via* purely carbon-based π-conjugated chains. In contrast, phosphonate-containing intramolecular hydrogen bonds remain underexplored,^12,13^ despite the ubiquity of phosphonates in synthesis (*e.g.*, HWE synthesis), medicinal chemistry, and bioactive molecules^14–16^ as well as the ongoing discussion on the nature of the phosphoryl bond,.^17–19^ The latter suggests that phosphoryl groups may support hydrogen bonds through a fundamentally different form of resonance assistance, namely negative hyperconjugation.

To address this gap, we investigate a family of phosphorylated hydroquinolinols (PHQs) A2(a), A3(a), A7(a), and A8(a) featuring rare intramolecular O–H⋯O=P hydrogen bonds closing a six-membered O2–P1–C1–C9–O1–H1 quasi-ring (Fig. 1). The compounds were prepared according to the previously published procedure.^12^ A Cambridge Structural Database (CSD)^20^ survey indicates that such intramolecular O–H⋯O=P synthons are very rare (only 13 deposited structures), and a systematic analysis of their structural and electronic features has not been reported. This study aims to characterize intramolecular O–H⋯O=P hydrogen bonds in the solid state and in solution, evaluate the extent of bond-length redistribution within the hydrogen-bond-closed quasi-ring, and examine the electronic interactions within the π-conjugated donor and phosphoryl acceptor regions. For this purpose, single-crystal X-ray (SCXRD) and neutron diffraction (for A7(a)),^21^ solution-state NMR and FT-IR spectroscopy, and NBO analysis with Wiberg bond indices were combined to assess whether the classical RAHB model adequately describes the observed hydrogen-bonding motifs or involves substantial contributions from alternative σ-type delocalization mechanisms.

**Fig. 1:**
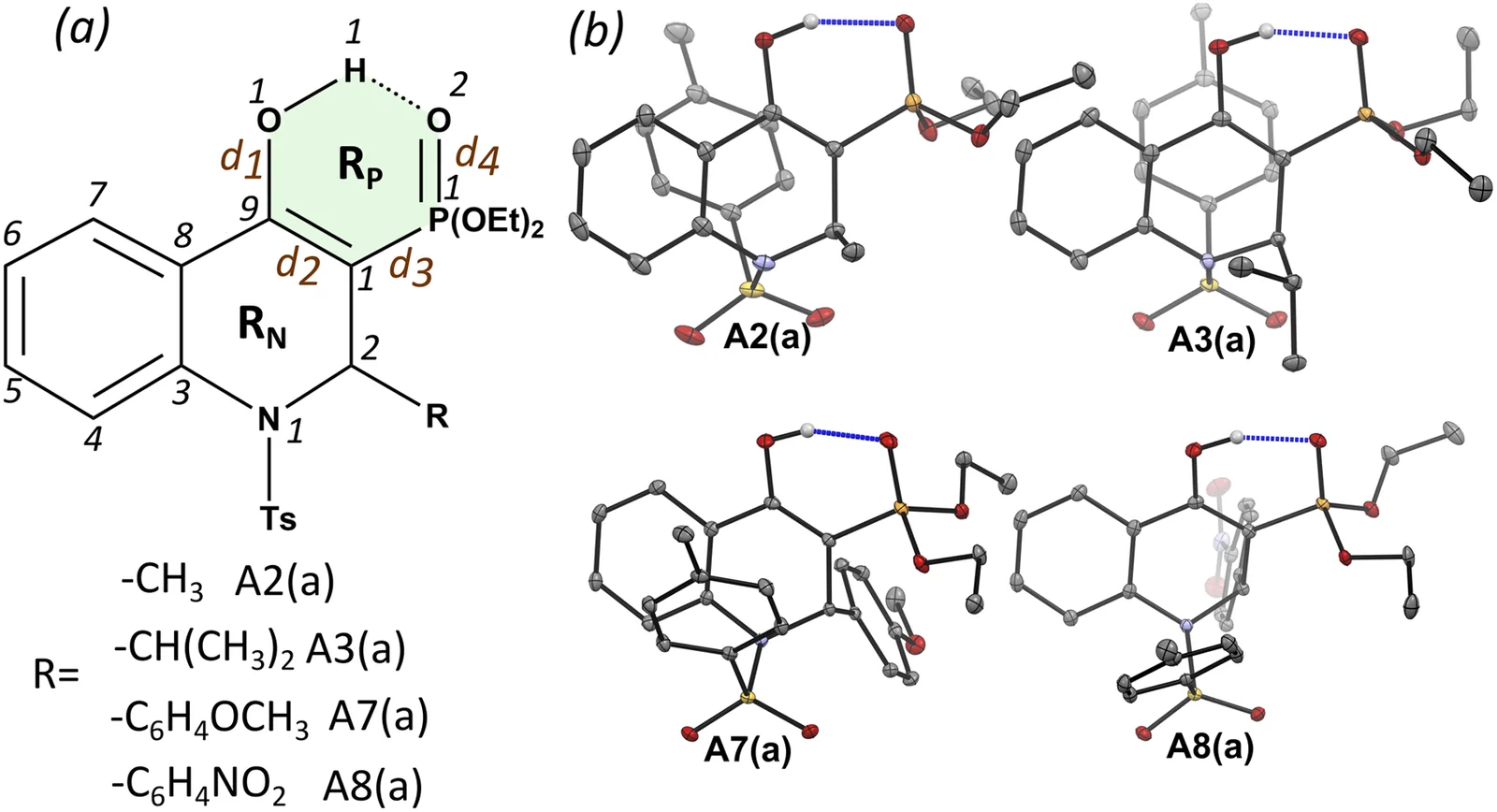
(a) Schematic representation of the intramolecular O–H⋯O=P hydrogen-bond synthon forming a six-membered quasi-ring (R_P_) fused with 4-hydroquinoline core (R_N_) and a numbering scheme for studied structures. (b) The molecular structures of A2(a), A3(a), A7(a), and A8(a) were determined by the SCXRD method, showing direct localization of the hydrogen atom involved in the intramolecular hydrogen bond, as determined for A7(a) by a neutron diffraction experiment (see SI). Remaining hydrogen atoms are not shown for clarity; thermal ellipsoids are drawn at the 50% probability level; a dashed line indicates the hydrogen bond. For A2(a), only one molecule from the asymmetric unit is shown for clarity.

## Results and discussion

### Solid-state structural characterization

To assess whether intramolecular O–H⋯O=P hydrogen bonds follow the classical RAHB paradigm or represent a distinct electronic regime, we first analyzed their structural characteristics. SCXRD revealed that the investigated PHQs share a common scaffold: a 4-hydroquinoline core (R_N_), *N*-tosylation (Ts) at N1 and diethoxyphosphoryl groups ((OEt)_2_) at P1, with C2 bearing either weak/moderate electron-donating groups (EDGs) (–CH_3_, –CH(CH_3_)_2_, –C_6_H_4_OCH_3_) or a strong electron-withdrawing nitrophenyl group (EWG) (–C_6_H_4_NO_2_). Since the substituents are attached to a saturated C2 carbon atom, any influence on the hydrogen-bonded motif is expected to arise primarily through σ-inductive, electrostatic, and steric effects rather than direct π-electronic communication.

In all structures, the hydroxy group at C9 (O1–H1) donates an intramolecular hydrogen bond to the phosphoryl oxygen O2, closing a six-membered quasi-ring *R*_P_=O2–P1–C1–C9–O1–H1 fused to the 4-hydroquinoline fragment (Fig. 1).

The O⋯O distances range from 2.601(1) to 2.684(2) Å (Table 1), with O–H bond lengths refined to ≈1.00–1.01(2) Å (neutron diffraction-derived H position of A7(a)_n_ is fixed for all X-ray structures), and H⋯O separations of 1.65–1.72(2) Å. These values are typical of strong, asymmetric intramolecular O–H⋯O hydrogen bonds, but are longer than the shortest O⋯O distances in archetypal β-dicarbonyl RAHBs (2.456–2.615 Å).^1^ Importantly, the neutron diffraction experiment resolves the fundamental ambiguity inherent to X-ray data: whether the observed geometry reflects a localized proton or a dynamically averaged hydrogen bond.

**Table 1:** Selected geometric parameters defining the intramolecular O–H⋯O=P hydrogen bond and the hydrogen-bond-closed quasi-ring in phosphorylated hydroquinoline derivatives A2(a), A3(a), A7(a), and A8(a), as determined by SCXRD and neutron diffraction methods

	O⋯O	O–H^a^	H⋯O	*d*_1_ C–O^b^	*d*_2_ C–C^c^	*d*_3_ C–P^d^	*d*_4_ P–O^e^	HOMA^f^
A2(a)_a_	2.669(2)	1.01(2)	1.71(2)	1.347(2)	1.356(2)	1.759(2)	1.481(1)	0.31
A2(a)_b_	2.684(2)	1.01(2)	1.72(2)	1.347(1)	1.356(2)	1.759(2)	1.484(2)	0.33
A3(a)	2.609(1)	1.01(1)	1.65(1)	1.347(2)	1.357(2)	1.764(1)	1.483(1)	0.31
A7(a)	2.601(1)	1.01(2)	1.64(2)	1.348(1)	1.356(1)	1.765(1)	1.482(1)	0.29
A8(a)	2.645(1)	1.01(2)	1.68(2)	1.346(1)	1.355(2)	1.763(1)	1.483(1)	0.31

a The value for A7(a) derives directly from a neutron diffraction experiment and was fixed in refinements of SCXRD structures (for details, see SI). Referenced bond length values are from international tables for crystallography.

b Values may be referenced to enol systems (C=C–OH, 1.333 Å), phenols (C_ar_–O, 1.362 Å), or “unperturbed geometry” (1.353 Å).

c 1.341 Å.

d 1.786 Å.

e 1.457 Å.

f Details on HOMA calculation see SI.

The presence of a hydrogen-bond-closed quasi-ring (R_P_) naturally invites comparison with classical RAHB systems. However, a closer examination of the bond-length pattern within R_P_ reveals deviations from the canonical RAHB paradigm and a limited degree of bond-length equalization (Table 1). The C9–O1 bond (*d*_1_ ≈ 1.346–1.348 Å) is markedly shorter than a typical phenolic C_ar_–O bond (1.362 Å), but only marginally shorter than the “unperturbed geometry” 1.353 Å value proposed for Gilli's RAHB reference geometry.^1^ The formally double C1–C9 and P1–O2 bonds (*d*_2_ and *d*_4_) are slightly elongated (∼1–2%), while the P1–C1 bond (*d*_3_) is modestly shortened (∼1.5%) relative to the single-bond reference. Overall, the bond-length distortions are clearly smaller than in β-dicarbonyl RAHBs, where deviations from reference values reach 4.7% for *d*_1_, 4.0% for *d*_2_, 2% for *d*_3_, and 5.8% for *d*_4_.^1^ Thus, while the geometries fall within the range of strong intraHBs, they do not display the degree of bond equalization expected for fully developed RAHB systems.

Estimation of delocalization in R_P_ using the Harmonic Oscillator Model of Aromaticity (HOMA)^22^ yields moderate values of 0.29–0.33 across the series (Table 1), significantly lower than HOMA ≈ 0.6, typical of classical RAHB quasi-rings,^23,24^ but comparable to quasi-rings fused with phenylene fragments (HOMA ≈ 0.30).^25^ We emphasize that HOMA, originally parameterized for C–C rings, is used here as a qualitative indicator of bond-length equalization rather than a strict aromaticity measure for O/P-containing rings (for details see SI) and provide a comparative metric. The moderate HOMA values, together with the elongated O⋯O distances, indicate partial π-delocalization and limited bond equalization, inconsistent with the extensive bond equalization characteristic of classical RAHB systems.

It is well known that aromaticity may also be quantified by an effective characteristic introduced by Schleyer *et al.* and further developed into the nucleus-independent chemical shift (NICS).^26^ However, it was shown that NICS poorly describes π-electron delocalization in quasi-aromatic rings. Because R_P_ is intrinsically heteronuclear, classical symmetry-based descriptors developed for homonuclear RAHB systems (such as Q or λ)^1,4^ cannot be meaningfully applied. The built-in asymmetry of the donor-to-acceptor linking chain underscores that, despite geometric similarities, the O–H⋯O=P synthon is not a straightforward analog of a canonical carbonyl-based RAHB. The reduced structural equalization observed here already suggests that the underlying electronic mechanism may differ from that of canonical RAHB interactions.

### Orbital interactions landscape

To elucidate the electronic origin of the O–H⋯O=P hydrogen bond and determine whether it follows the classical RAHB mechanism or a distinct stabilization regime, we performed a detailed NBO with Wiberg bond indices (WBIs) analysis.^27^ The system was conceptually partitioned into donor and acceptor regions to differentiate between π-delocalization within the hydroquinolinol framework and σ-type interactions associated with the phosphoryl group (Fig. 2).

**Fig. 2:**
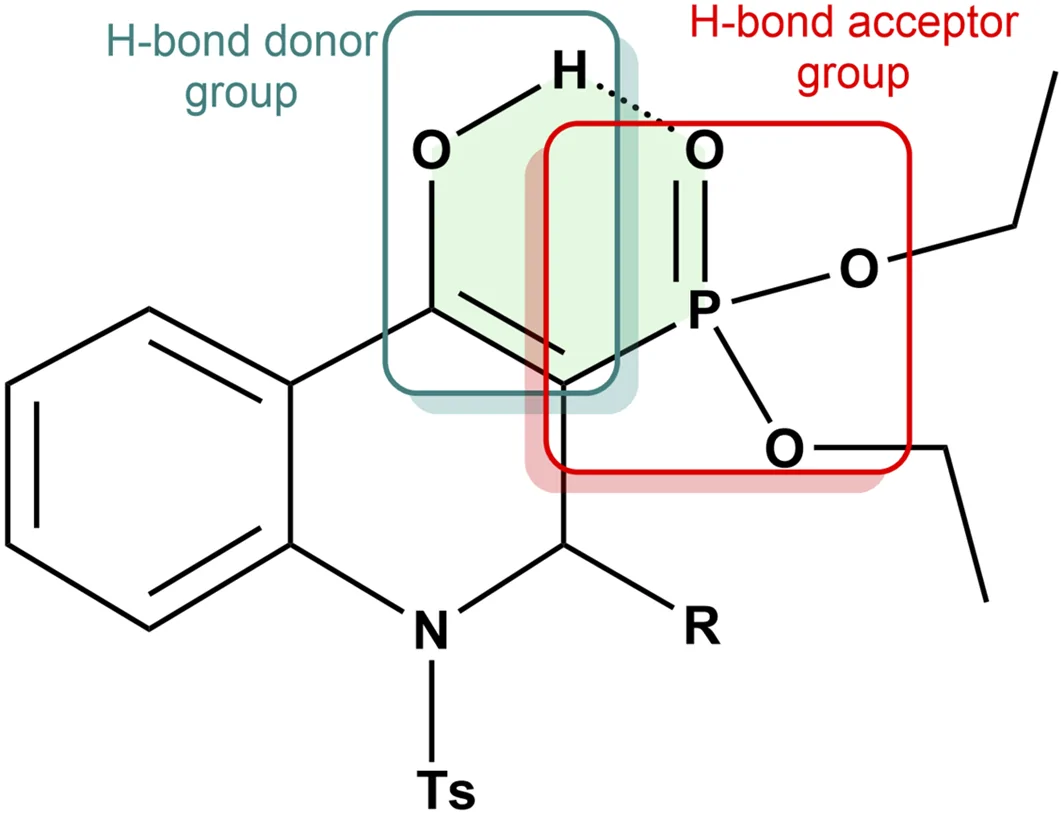
Conceptual boundaries delineate interpenetrating regions affecting the donor–acceptor properties of a given system.

On the acceptor side, the phosphoryl group is stabilized predominantly by negative hyperconjugation involving the O2 lone pairs and antibonding σ* orbitals of P–O and P–C bonds. It is in accordance with the contemporary concept of the nature of the phosphoryl group.^17–19^ The key interactions *n*(O2) → σ*(P1–O3), *n*(O2) → σ*(P1–O4), and *n*(O2) → σ*(P1–C1) contribute stabilization energies of ∼20 kcal mol^−1^ for *n*(O) → σ*(P–O) and ∼10 kcal mol^−1^ for *n*(O) → σ*(P–C), with total *n*(O2) → σ*(P–X) contributions of *ca.* 65 kcal mol^−1^ nearly invariant across the series (Table 2). The remarkable invariance of these interactions is consistent with a buffering role of the negative-hyperconjugative phosphoryl acceptor domain across the investigated substitution series. Additionally, negative hyperconjugation is clearly supported by *n*(O2) → Rydberg(P) delocalization, with stabilization energies of 3.4–7.4 kcal mol^−1^. These interactions reflect the pronounced σ-type acceptor character of the phosphorus center and may be regarded as a manifestation of *n* → d-type delocalization, insofar as the acceptor Rydberg orbitals are dominated by basis functions of *d*-type angular character. This description should be understood in a formal, basis-set–dependent sense and reflects the polarization and charge-accepting capacity of the phosphorus atom rather than the direct involvement of valence d orbitals in bonding. Although the dominant donor–acceptor interactions involve Rydberg(P) rather than σ(P1–O2) orbitals, the overall electronic features of the P–O bond are consistent with a σ-resonance/negative hyperconjugation framework.

**Table 2:** Selected NBO second-order perturbation stabilization energies (*E*(2), kcal mol^−1^) for donor–acceptor interactions associated with the intramolecular O–H⋯O=P hydrogen bond in compounds A2(a), A3(a), A7(a), and A8(a). Interactions are sorted by their function within the structure. For more detailed information, see SI

Donor NBO → acceptor NBO	A2(a)_a_	A2(a)_b_	A3(a)	A7(a)	A8(a)
Negative hyperconjugation *n* → σ*/intraHB stabilization					
*n*_1_(O2) → σ* (O1–H1)	4.15	3.94	5.33	5.61	4.61
*n*_2_(O2) → σ*(O1–H1)	7.09	2.18	1.14	9.28	2.20
*n*_3_ (O2) → σ*(O1–H1)	12.83	17.55	23.22	16.18	20.83
Total	24.07	23.77	29.69	31.07	27.64

Hyperconjugation: σ → σ*/O–H bond destabilization					
σ(O1–H1) → σ*(C8–C9)	4.97	5.13	4.85	4.91	5.17

Negative hyperconjugation *n*/π → σ*/P-center stabilization					
*n*_1_(O2) → σ*(P1–C1)	1.11	1.13	1.20	1.22	1.13
*n*_2_(O2) → σ*(P1–C1)	7.75	3.19	1.97	4.95	3.55
*n*_3_(O2) → σ*(P1–C1)	6.75	10.95	11.85	8.98	10.69
*n*_2_(O2) → σ*(P1–O3)	23.64	25.04	24.20	2.97	24.91
*n*_3_(O2) → σ*(P1–O3)	1.36	<0.5	1.03	21.94	<0.5
*n*_2_(O2) → σ*(P1–O4)	0.70	5.64	8.03	24.84	5.37
*n*_3_(O2) → σ*(P1–O4)	24.55	19.35	16.54	<0.5	19.59
π(C1–C9) → σ*(P1–O3)	5.31	3.76	4.07	3.41	4.81
π(C1–C9) → σ*(P1–O4)	3.69	5.12	5.20	5.37	4.29
Total	74.86	74.18	74.09	73.68	74.34

***n*–-d/p(P) delocalization/P-center stabilization**					
*n*_1_(O2) → RY_6_(P1)	5.97	5.78	5.78	5.68	6.00
*n*_2_(O2) → RY_1_(P1)	5.07	6.54	7.38	4.61	7.10
*n*_3_(O2) → RY_2_(P1)	4.81	5.94	6.71	3.39	6.65

Conjugation *n*→π*/electron delocalization over R_**N**_ **– ring**					
*n*_2_(O1) → π*(C1–C9)	42.86	42.65	43.84	43.88	44.42
π(C3–C8) → π*(C1–C9)	16.65	16.26	16.70	16.96	17.79
*n*_2_(N1) → π*(C3–C8)	7.92	8.44	9.91	10.13	15.21
Total	67.43	67.35	70.45	70.97	77.42

Hyperconjugation σ → σ* and negative hyperconjugation *n*/π → σ*/electron delocalization over R_**N**_ – **ring**					
*n*_1_(O1) → σ*(C1–C9)	6.17	6.11	6.29	6.34	6.00
π(C1–C9) → σ*(C3–C8)	9.70	9.76	9.37	9.22	8.66
*n*_1_(N1) → σ*(C3–C8)	5.30	5.16	5.12	4.86	5.91
*n*_3_(N1) → σ*(C1–C2)	5.64	5.60	4.93	4.85	3.57
σ(C1–C2) → σ*(O1–C9)	5.79	5.76	5.47	5.42	5.42
Total	32.6	32.4	31.2	30.7	29.6

WBIs (Table 3) confirm that the formally double P1–O2 bond is weakened (WBI ≈ 1.08), P–O(ethoxy) bonds are significantly polarized (WBI < 1.0), and the P1–C1 bond (WBI ≈ 0.94) behaves as a polarized single bond. Overall, the phosphoryl fragment acquires a quasi-ionic character with a partially positive phosphorus and a negatively charged phosphoryl oxygen, creating a strongly polarized intraHB acceptor region.

**Table 3:** Properties of average values of Wiberg bond order (WBI). Detailed individual values for all structures data in SI

Bond	WBI	Bond	WBI
P1–O2	1.080(2)	O1–H1	0.801(4)
P1–C1	0.936(2)	P1–O3	0.866(1)
C1–C9	1.269(3)	P1–O4	0.857(3)
C9–O1	1.048(3)	H1⋯O2	0.280(13)

Accordingly, the strong delocalization originating from second-order conjugation^28^ (*i.e.*, hyperconjugation and negative hyperconjugation) results in σ-resonance.^29^ Indeed, hyperconjugation has already been shown to support intraHB formation in saturated systems.^30^ However, this effect was also raised to undermine the RAHB paradigm by stating that, in saturated systems, the strength of intraHB follows the rigid σ-skeleton.^31^ We clearly observe that the sum of orbital interactions following the negative hyperconjugation over the H-bond acceptor group remains stable regardless of the substituent type at the C2 atom. The strong effect of negative hyperconjugation suggests that the formed O–H⋯O=P intraHB follows the resulting geometry rather than resonance in Gilli's term, being in accordance with a voice of criticism raised by Alkorta *et al.*^32,33^

On the donor side, the hydrogen bond donor group consists of an enol group –OH linked to a six-membered *N*-containing ring (R_N_) of the quinolinol fragment, characterized by a system of conjugated CC bonds. The strongest stabilization (*E*(2) = 42.6–44.4 kcal mol^−1^) arises from classical (first-order) Mulliken conjugation^28^*n*(O1) → π*(C1–C9) (WBI ≈ 1.3), showing a major π-resonance contribution and extensive delocalization across the quinoline moiety (Fig. 3). Additional conjugation effects π(C3–C8) → π*(C1–C9) and *n*(N1) → π*(C3–C8) give ∼17 kcal mol^−1^ and ∼7.9–15.2 kcal mol^−1^ stabilization, respectively. These classical conjugation effects are reinforced by σ → σ* and *n*/π → σ* hyperconjugation interactions, which enhance delocalization in R_N_. Notably, the central *n*(O1) → π(C1–C9) conjugative interaction remains nearly invariant across the investigated substitution series, with *E*(2) values of 42.65–44.42 kcal mol^−1^.

**Fig. 3:**
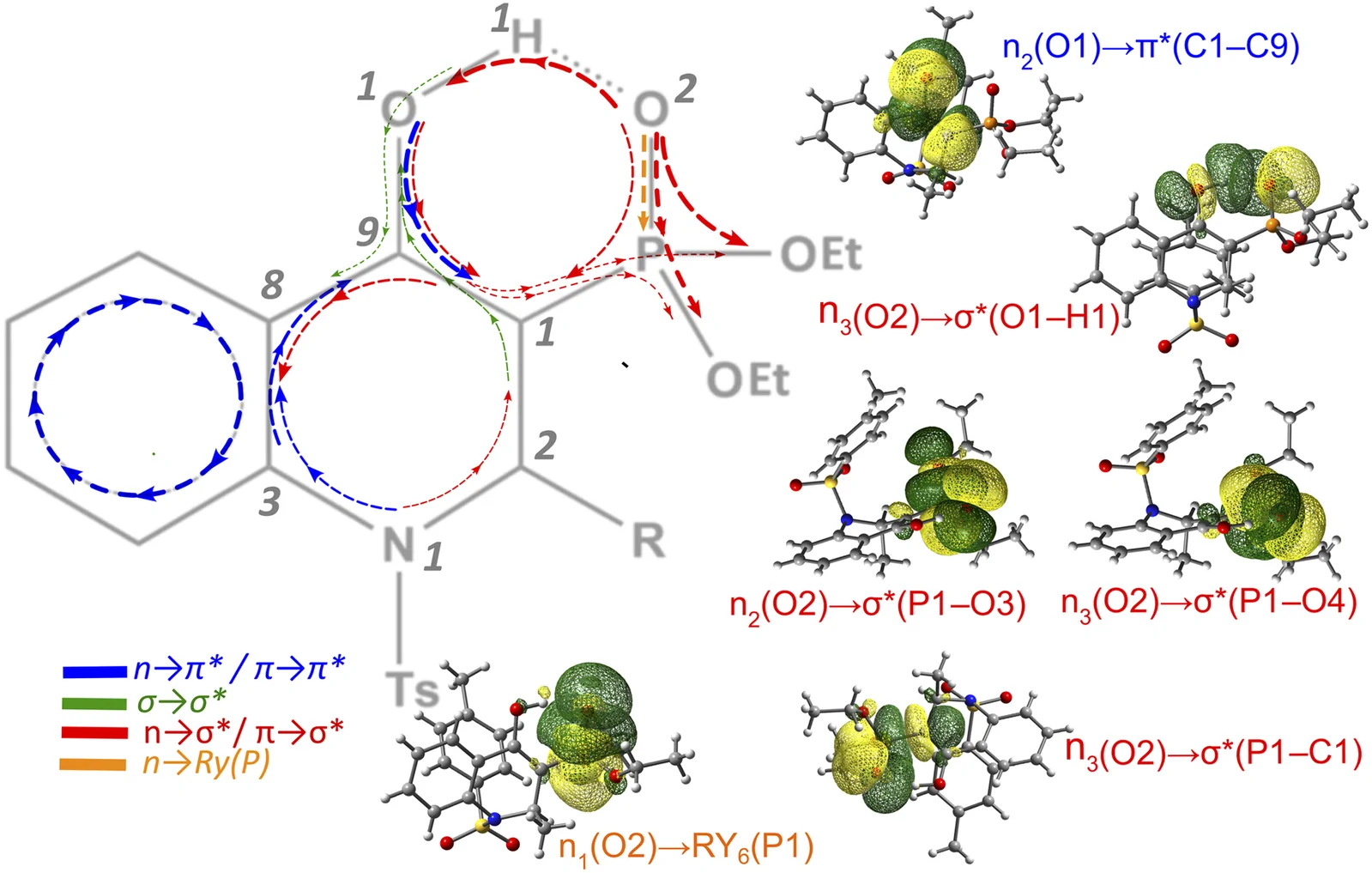
Schematic representation of major components of electron delocalization with selected NBO orbital visualization: blue-classical (first-order) conjugation, green – hyperconjugation, red – negative hyperconjugation, orange – *n* → *d*(P) delocalization. Line width is proportional to the strengths of relevant orbital interactions. Visualized orbitals refer to the structure A2(a). For detailed visualization of the orbital interactions, see Fig. S6–S10.

Electron transfer *n*(O1) → π*(C1–C9) decreases the localized π-character of C1–C9 (WBI ≈ 1.27), strengthens C9–O1 (WBI ≈ 1.05), and weakens O1–H1 (WBI ≈ 0.8), making the enol a strong H-bond donor. NBO analysis shows that classical conjugation strongly influences the donor region, with π-resonance dominating over σ-resonance in stabilizing the H-bond donor domain. No correlation is observed between total orbital interaction energies of intraHB O–H⋯O=P formation and the relevant energies of orbital interactions following π- or σ-resonance within the quasi-ring.

The intramolecular hydrogen bond is stabilized by electron transfer from the O2 lone pairs into the σ*(O1–H1) antibonding orbital. The cumulative *n*(O2) → σ*(O1–H1) stabilization ranges from ∼24 to ∼31 kcal mol^−1^, with the largest contributions originating from the *n*_3_(O2) lone pair (12.8–23.2 kcal mol^−1^). The intraHBs are characterized by WBIs of ≈0.28 for H1⋯O2 and ≈0.80 for O1–H1, consistent with strong yet clearly asymmetric O–H⋯O hydrogen bonds rather than symmetric low-barrier interactions.^34^

Importantly, the total stabilization associated with the *n*(O2) → σ*(O1–H1) interaction shows no systematic dependence on substituent electronic properties. For example, both moderately (–CH(CH_3_)_2_) and strongly (–C_6_H_4_OCH_3_) electron-donating substituents yield high stabilization energies (*E*(2) = 29.7 and 31.1 kcal mol^−1^, respectively), whereas a weak donor (–CH_3_) gives a lower value (∼24 kcal mol^−1^), and a strong electron-withdrawing group (–C_6_H_4_NO_2_) results in an intermediate stabilization (∼28 kcal mol^−1^). Although variations of ∼7 kcal mol^−1^ are observed, these changes are irregular and do not follow a consistent substituent-dependent trend. Notably, although the geometries of symmetry-independent molecules A2(a)_a_ and A2(a)_b_ differ slightly, the associated *n*(O2) → σ*(O1–H1) stabilization energies remain comparable.

Within the investigated series, the key hydrogen-bond descriptors show limited and non-systematic variation. This behavior indicates an attenuated response to the structural modifications examined here, although its exact molecular origin cannot be established unambiguously because the substituents are not directly conjugated with the π-system. Consequently, the present series should not be regarded as a classical π-substituent study. Instead, it probes the response of the O–H⋯O=P interaction to the σ-electronic and steric perturbations.

Thus, the investigated systems are not devoid of π-resonance. Rather, they exhibit an electronically differentiated, mixed π/σ regime in which substantial π-delocalization occurs primarily within the donor domain, while σ-type negative hyperconjugation makes a major contribution within the phosphoryl acceptor domain. These electronically distinct delocalization mechanisms cooperate to sustain the intramolecular hydrogen bond; consequently, the classical cooperative RAHB model does not fully account for the electronic stabilization of the complete O–H⋯O=P quasi-ring.

### Spectroscopy of IntraHB O–H⋯O=P in solution

The presented results are based on observations and molecular geometries in crystal environments. To track the behavior of O–H⋯O=P intraHB in liquids, we recorded ^1^H and ^31^P NMR spectra for A2(a), A3(a), A7(a), and A8(a) in CD_3_CN (Fig. S12–S15). Singlet signals at ∼11 ppm (10.84 ppm, 11.05 ppm, 11.10 ppm, and 11.14 ppm, respectively) originate from the proton engaged in an intramolecular hydrogen bond. These values are downfield from resonances typical of aromatic hydroxyl groups (Ar–OH) and clearly higher than literature chemical shifts for intermolecular Ar–OH⋯O=P hydrogen bonds in protein–DNA complexes (∼9.7–9.9 ppm).^35^

The C2-substitution exerts a moderate effect on ^31^P and ^1^H NMR chemical shifts. EDGs shift phosphorus downfield, whereas EWG in A8(a) produces the lowest ^31^P shift, indicating strong shielding. For the O–H⋯O=P proton, ^1^H shifts increase with substituent size and electron-withdrawing character, reaching 11.14 ppm for the nitrophenyl derivative. However, the relatively small shift range (10.84–11.14 ppm) shows that C2-substituents negligibly modulate electron density and deshielding of the involved nuclei. The results suggest that the structural modifications introduced at the C2 position exert only a minor influence on the spectroscopic descriptors of the intramolecular hydrogen bond within the investigated series. This observation is consistent with the NBO results.

### IntraHB persistence in solution

The persistence and relative strength of the O–H⋯O=P intraHB in solution were examined by concentration-dependent ^1^H NMR spectroscopy in deuterated dimethyl sulfoxide (C_2_D_6_SO), a strongly HB-accepting solvent commonly used to probe the robustness of intramolecular hydrogen bonds (Fig. 4). Accordingly, the intensity of the ∼11 ppm signal decreases upon dilution and, in some cases, vanishes at high dilution (A3(a) and A8(a)). The OH resonance also shows non-monotonic linewidth variations across the concentration series, which may reflect proton exchange with residual water or transiently DMSO-solvated states. However, the lowest-concentration spectra were recorded using solvent and water presaturation and had lower signal-to-noise ratios; therefore, their line shapes are not directly comparable with those obtained using the standard ^1^H NMR experiment. We consequently do not interpret the linewidth variations as a quantitative measure of hydrogen-bond strength. The essentially unchanged OH chemical shift over the concentration range in which the signal is reliably detectable is consistent with persistence of the intraHB, although rapid exchange with solvent- or water-associated states cannot be excluded.

**Fig. 4:**
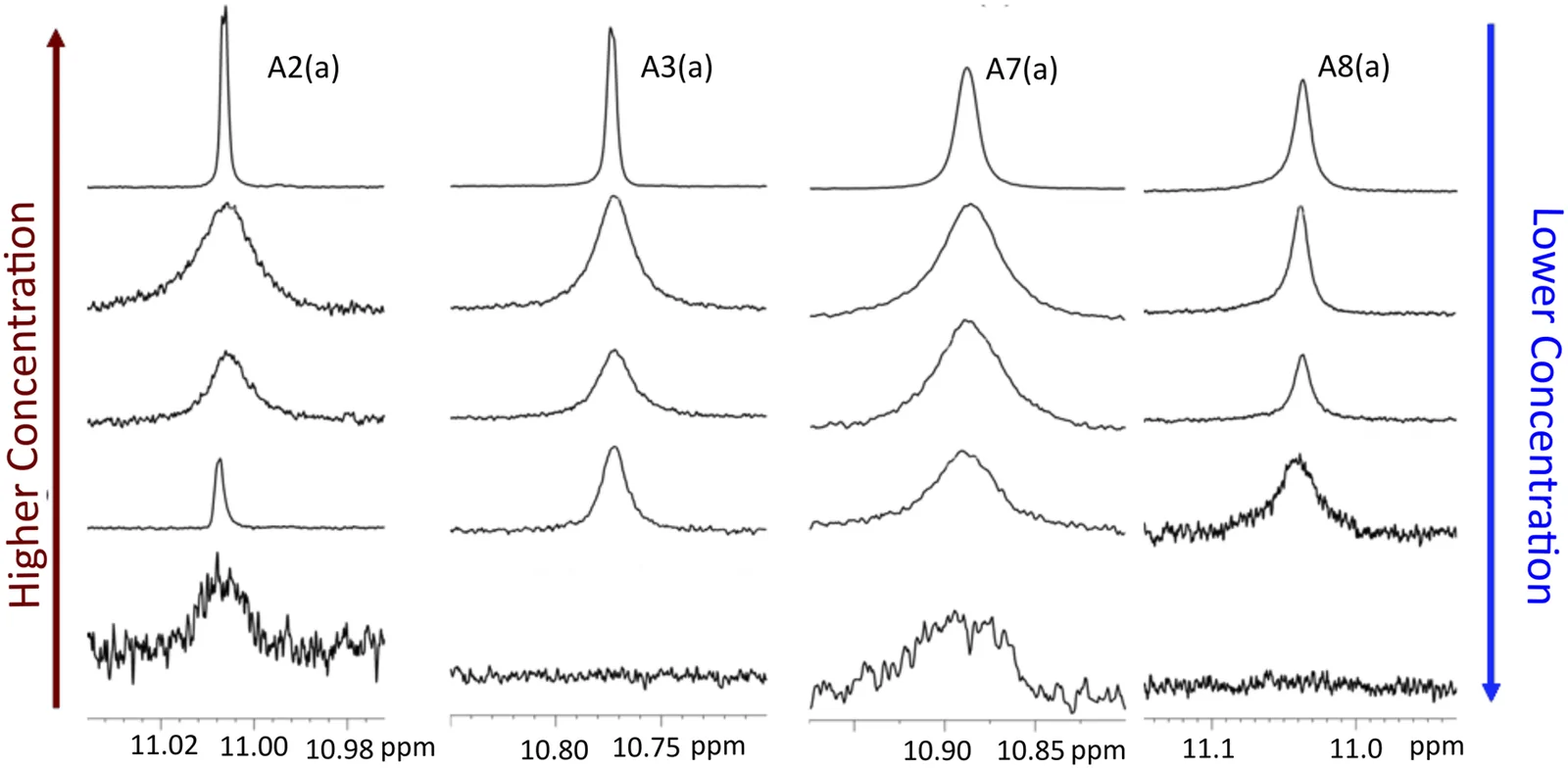
Signals originating from the proton involved in the intramolecular hydrogen bond for different concentrations in C_2_D_6_SO.

An independent empirical estimate based on Abraham's A_NMR_ parameter (the difference in chemical shift of the hydrogen-bonded proton measured in CDCl_3_ and C_2_D_6_SO; for details, see SI)^36,37^ yields values close to zero, well below the 0.1 threshold, and thus indicates exceptionally strong intramolecular hydrogen bonds. FTIR data (see SI) fully support the NMR findings. The redshift of the O–H band to 3200–2700 cm^−1^ and its identical position in solid and liquid phases confirm a moderate-to-strong intramolecular O–H⋯O=P hydrogen bond. The results confirm that C2-substituents do not disrupt the robust intraHB.

The qualitative agreement among solid-state structural metrics, NBO stabilization energies, and solution-state NMR parameters demonstrates that the O–H⋯O=P interaction is intrinsic and persistent, rather than an artifact of crystal packing. We note that NBO-derived stabilization energies and NMR-based descriptors probe distinct physical aspects of the hydrogen bond and are therefore unlikely to correlate quantitatively. Nevertheless, both approaches consistently identify O–H⋯O=P synthon as strong and persistent intraHBs with a stabilization mechanism dominated by local electronic effects, rather than long-range cooperative π-delocalization, reinforcing the conclusion that the intraHB is not governed by a classical RAHB-type mechanism.

Taken together, these results reveal a clear separation between the geometric features traditionally associated with RAHB and the underlying electronic mechanism stabilizing the hydrogen bond. While the O–H⋯O=P motif adopts geometries reminiscent of classical RAHB systems, its stabilization arises from a fundamentally different balance of interactions, dominated by localized hyperconjugative effects at the phosphoryl acceptor. These findings demonstrate that RAHB-like structural signatures do not necessarily imply a common electronic origin, and that strong intramolecular hydrogen bonds can be sustained when cooperative π-resonance is limited and is not the dominant factor controlling hydrogen-bond strength within the investigated series.

## Conclusions

In summary, we demonstrate that intramolecular O–H⋯O=P hydrogen bonds constitute a distinct electronic regime beyond classical RAHB. Although these interactions adopt geometries reminiscent of RAHB systems, their structural signatures are not accompanied by the extensive π-electron delocalization characteristic of canonical resonance-assisted hydrogen bonds.

Combined crystallographic, NBO, and NMR analyses confirm that these interactions are strong and exhibit apparent resonance-like signatures, manifested by cooperative donor–acceptor delocalization, measurable bond-length redistribution within the quasi-ring, and pronounced downfield ^1^H shifts. However, their limited bond equalization and mixed π/σ-resonance origin clearly distinguish them from archetypal π-delocalized β-dicarbonyl RAHB systems. A substantial contribution of σ-type hyperconjugative delocalization over the phosphoryl center, the weak dependence of hydrogen-bond strength on substituent effects, and the absence of a systematic relationship between the hydrogen-bond descriptors and the π-domain parameters indicate that classical cooperative π-resonance is not the only dominant factor controlling variations in These findings show that RAHB-like geometries may arise from fundamentally different electronic origins and that strong intramolecular hydrogen bonds do not necessarily require extended π-conjugation. Instead, hyperconjugation-driven stabilization can sustain robust hydrogen bonding in heteronuclear frameworks, including systems containing saturated fragments. This establishes a design principle for intramolecular hydrogen bonds displaying a buffered response to the σ-electronic and steric perturbations represented within the investigated substitution series, opening new possibilities for controlling molecular preorganization and reactivity in phosphorus-containing systems and beyond.

Extending this approach to structurally diverse phosphonate frameworks and other related X=O motifs defined by negative hyperconjugation effects will enable a deeper exploration of how σ- and π- delocalization cooperate in shaping hydrogen-bond-driven reactivity and molecular preorganization.

## Author contributions

A. P. and W. M. W. supervised the project. A. P. acquired funding, conducted SCXRD and neutron diffraction experiments, NBO calculations, and data analysis. P. N. and M. K.D. conducted NMR and IR experiments and data analysis. S. C. C. conducted neutron diffraction measurements, data analysis, and validation. All. The authors contributed to writing, reviewing, and editing the manuscript and gave approval to the final version.

## Conflicts of interest

There are no conflicts to declare.

## Supplementary Material

RA-OLF-D6RA09698K-s001

## Data Availability

Supporting information (SI) contains all experimental procedures and associated data details on: single-crystal X-ray and neutron diffraction analysis, HOMA parameters, NBO calculations, NMR, and IR spectroscopy.^22,27,35,37–52^ Supplementary information is available. See DOI: https://doi.org/10.1039/d6ra09698k. CCDC 2546397 (for A2(a)), 2546396 (for A3(a)), 2546395 (for A7(a)), 2546394 (for A8(a)), and 2547090 (for A7(a)_n_) contain the supplementary crystallographic data for this paper.
