# Supplementary material for: When phosphorus takes over: hyperconjugation-driven intramolecular O–H⋯O[double bond, length as m-dash]P hydrogen bonds beyond classical RAHB
Source: RSC Adv. 2026 Nov 10. Online ahead of print. doi: 10.1039/d6ra09698k (PMC13749440; doi:10.1039/d6ra09698k)
Supplement: Supplementary file 1 [file RA-OLF-D6RA09698K-s001.pdf]

Supplementary Information (SI) for RSC Advances.
This journal is © The Royal Society of Chemistry

Supporting Information

for

When Phosphorus Takes Over: Hyperconjugation-Driven Intramolecular O-H···O=P Hydrogen Bonds Beyond Classical RAHB

Anna Pietrzak[a] , Przemysław Nowak[b,c], Silvia C. Capelli[d], Marta K. Dudek[b], Wojciech M. Wolf[a]*

[a] Dr. A. Pietrzak, Prof. W. M. Wolf
Institute of General and Ecological Chemistry, Faculty of Chemistry
Łódź University of Technology Żeromskiego 114, 90-543 Łódź
E-mail: anna.pietrzak.1@p.lodz.pl

[b]P. Nowak, Prof. M. K. Dudek
Centre of Molecular and Macromolecular Studies
Polish Academy of Sciences
Sienkiewicza 112, 90-363 Łódź, Poland

[c]P. Nowak
BioMedChem Doctoral School of University of Lodz and Lodz Institutes of the Polish Academy of Sciences
University of Łódź, Matejki 21/23, 90-237 Łódź, Poland

[d]Dr. S. C. Capelli
ISIS Neutron and Muon Facility Rutherford Appleton Laboratory
Didcot, OX11 0QX, United Kingdom

Table of Contents

S1. Single Crystal X-ray Diffraction Analysis (SCXRD)

S2. Neutron Diffraction Analysis

S3. HOMA Analysis

S4. NBO Calculations

S5. NMR Analysis

S6. FTIR Analysis

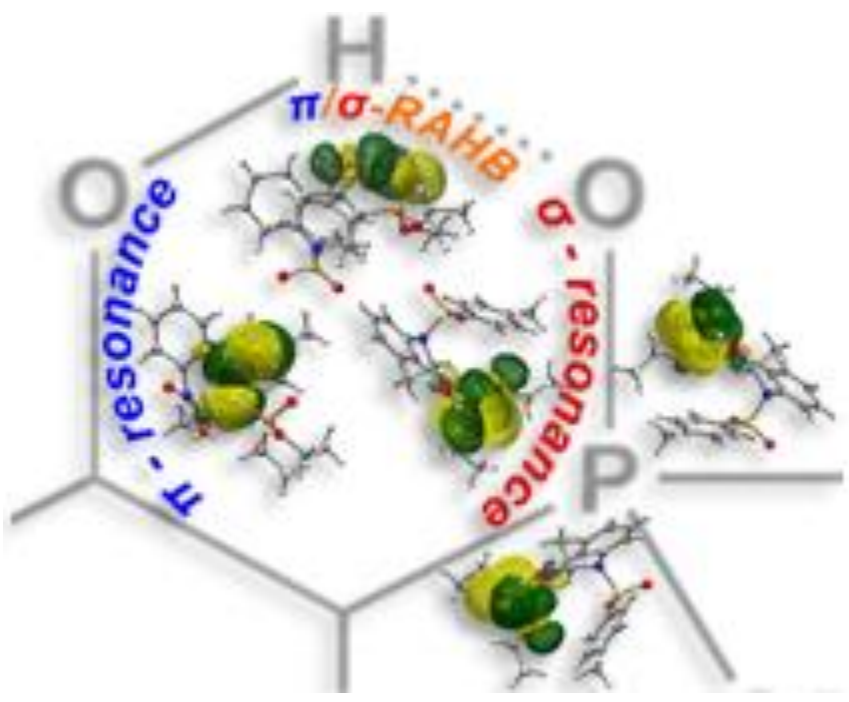

S1. Single Crystal X-ray Diffraction Analysis (SCXRD)

a) General comments

Crystals of **A2(a)** suitable for single-crystal X-ray diffraction (SCXRD) were obtained directly from the synthesis, while crystals of **A3(a)**, **A7(a)**, and **A8(a)** were obtained by slow evaporation. Structures **A3(a)** and **A8(a)** recrystallized from saturated solution in ethanol, while in **A7(a),** acetonitrile was used. All crystallization sets were kept in the fridge for approximately 2 weeks. The SCXRD data were collected using a dual-source Rigaku XtaLAB Synergy Pilatus 300K diffractometer at 100.0(1) K with a microfocus X-ray source and Cu Kα radiation (λ = 1.54184 Å). The data were integrated using CrysAlisPro.[1] Intensities for absorption were corrected using gaussian method as in SCALE3 ABSPACK scaling algorithm implemented in CrysAlisPro program. The crystal data and structure refinement descriptors for all structures are listed in Table S1.

Files CCDC 2546394–2546397 contain supplementary crystallographic data for this paper. These data can be obtained free of charge from the Cambridge Crystallographic Data Centre via www.ccdc.cam.ac.uk/structures.

Table S1. Crystal data and structure refinement parameters for **A2(a)**, **A3(a)**, **A7(a)**, and **A8(a)**.

**Parameter**<br>**CCDC number**	**A2(a)**<br>**2546397**	**A3(a)**<br>**2546396**	**A7(a)**<br>**2546395**	**A8(a)**<br>**2546394**
Empirical formula	$C_{21}H_{26}NO_6PS$	$C_{23}H_{30}NO_6PS$	$C_{27}H_{30}NO_7PS$	$C_{26}H_{27}N_2O_8PS$
Formula weight	451.46	479.51	543.55	558.52
Crystal system	Triclinic	Monoclinic	Monoclinic	Triclinic
Space group	$P\bar{1}$	$P2_1/n$	$P2_1/n$	$P\bar{1}$
a / Å	10.5517(2)	9.2048(2)	10.703(2)	8.7017(2)
b / Å	14.4734(3)	19.4487(2)	20.6165(3)	10.11610(10)
c / Å	16.4385(2)	13.3777(3)	12.0047(2)	16.0889(2)
α / °	64.579(3)	90	90	93.901(2)
β / °	83.184(2)	90.034(2)	99.437(4)	95.5150(10)
γ / °	71.998(2)	90	90	114.151(2)
V / Å³	2156.12(9)	2394.89(8)	2613.1(5)	1277.30(4)
Z	4	4	4	2
ρ_{calc} / g·cm⁻³	1.391	1.330	1.382	1.452
μ / mm⁻¹	2.366	2.160	2.081	2.189
2Θ range for data collection / °	5.954 to 148.996	8.022 to 140.146	8.578 to 158.506	5.558 to 148.972
Index ranges	-13 ≤ h ≤ 13, -17 ≤ k ≤ 18, -20 ≤ l ≤ 20	-11 ≤ h ≤ 11, -23 ≤ k ≤ 23, -15 ≤ l ≤ 16	-13 ≤ h ≤ 12, -26 ≤ k ≤ 26, -14 ≤ l ≤ 15	-10 ≤ h ≤ 10, -12 ≤ k ≤ 12, -20 ≤ l ≤ 20
Refl. collected	85365	22814	53925	49729
Independent reflections (R_{int})	8747 (0.0320)	4459 (0.0252)	5630 (0.0235)	5171 (0.0243)
$R1$, w$R2$ [$I > 2\sigma(I)$]	0.0322, 0.0881	0.0287, 0.0758	0.0290, 0.0772	0.0288, 0.0755
$R1$, w$R2$ (all data)	0.0332, 0.0888	0.0297, 0.0756	0.0293, 0.0775	0.0289, 0.0756
GOF on F^2	1.032	1.026	1.030	1.037
Largest diff. peak/hole / e·Å⁻³	0.51/-0.53	0.39/-0.41	0.33/-0.46	0.33/-0.40

b) structure solution and refinement

The structures were solved using SHELXT[2] by intrinsic phasing and refined by full-matrix least-squares minimization on F^2 using SHELXL[3], as implemented in ShelXle[4]. All non-hydrogen atoms were refined anisotropically. In the SCXRD models of all investigated structures (**A2(a)**, **A3(a)**, **A7(a)**, and **A8(a)**), the position of the hydrogen atom H1 involved in the O1–H1 bond was fixed using an O1–H1 distance corresponding to that determined from a neutron diffraction experiment performed for structure **A7(a)$_n$** (see Chapter S2. Neutron Diffraction Analysis). For this purpose, relevant DFIX and DANG restraints were used. All remaining hydrogen atoms were placed in calculated positions and included in the refinement using a riding model. One ethoxy group in a molecule of **A2(a)** is disordered over two sites, with an occupancy ratio of 0.53(2):0.47(2). To separate disordered atoms, the PART instruction has

been applied. To maintain reasonable ellipsoids (ADPs), relevant restraints, such as SIMU, RIGU, and DELU, were applied to relevant atoms.

c) Description of the crystal structures

Structures of **A2(a)**, **A8(a)** crystallize in triclinic *P*-1 spacegroup, while **A3(a)** and **A7(a)** form monoclinic systems *P*2$_1$/n. Centrosymmetric space groups indicate that crystals are racemates of *R* and *S* forms of molecules with a chiral center at the C2 atom. The crystal structure of **A2(a)** is formed by two symmetrically independent molecules **A2(a)_a** and **A2(a)_b**. Molecular structures of the systems are defined by the 4-hydroquinoline moiety linked to a tosyl group (Ts) at the nitrogen atom (N1) and a diethoxyphosphoryl group linked to the C1 atom. Structures **A2(a)**, **A3(a), and A4(a)** are substituted at the C2 atom by electron donor groups (EDG) from neutral/weak -CH_3 and -$CH(CH_3)_2$, respectively, to stronger **A7(a)** – $C_6H_4OCH_3$, while **A8(a)** is substituted by a strong electron acceptor group (EWG) -$C_4H_6NO_2$. Relevant molecular structures determined by SCXRD analysis are shown in Figures S1 and S2. The pyridine fragment fused with the benzene fragment adopts a slightly twisted conformation in all structures. However, the conformational freedom is limited by the ring's unsaturated fragments. The screw is evidenced by a weighted-average absolute torsion angle for the C1-C2-N1-C3-C8-C9 ring, ranging from 22.2 to 26.4°. Significant deviations of atoms C2 and N1 from the mean plane are defined by absolute torsion angles C1-C2-N1-C3 ranging from 41.6(1) to 48.5(2)°. Substituents at the C2 atom in all systems adopt an axial position, while the phosphoryl fragment is linked with C1 in an equatorial setting. The spatial orientation of the tosyl group linked to the N1 atom in both molecules of **A2(a)** is defined by the angle between the C2-C21 bond and the mean plane of C1-C2-N1-C3-C8-C9 ring of ~25°, corresponding to values characteristic of an axial position. In **A3(a)**, **A7(a),** and **A8(a)**, the value increases slightly, exceeding 30°, approaching the bisectorial position. Relevant nond lengths and angles are given in Tables S2-S12. In all structures, the phosphonate center adopts a tetrahedral geometry, with the phosphoryl bond (P=O) coplanar with the enol group -OH at the C9 atom. The formed intramolecular hydrogen bond closes a *quasi*-ring composed of O2, P1, C1, C2, C9, O1, and H1 atoms.

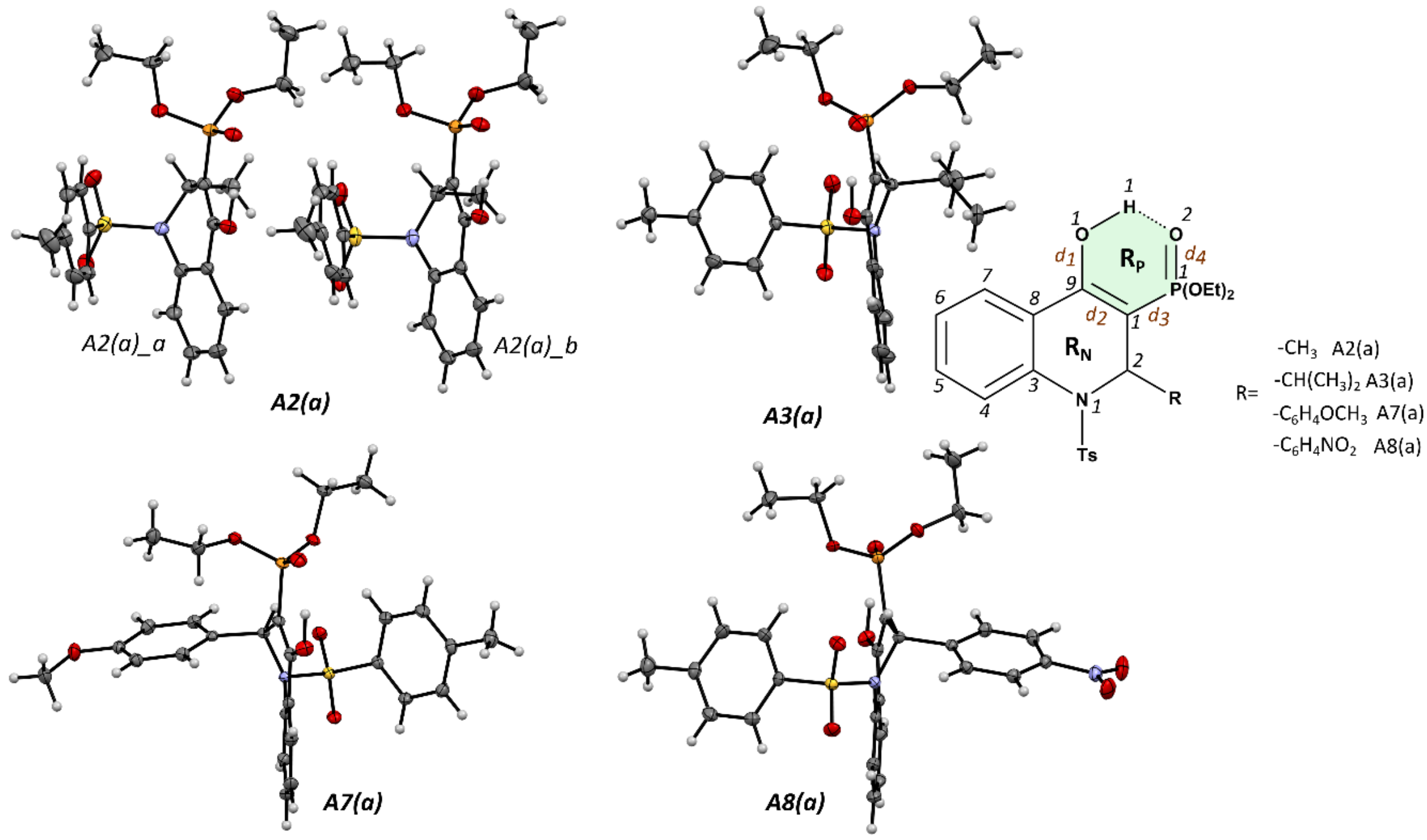

Figure S1. Moleluclar structures and atom numbering scheme of **A2(a)**, **A3(a)**, **A7(a)**, and **A8(a)**. Only one disorder group in molecule **A2(a)_b** is shown for clarity. Atomic displacement ellipsoids (ADPs) are drawn at 50% probability level. Hydrogen atoms are shown as spheres of arbitrary radii.

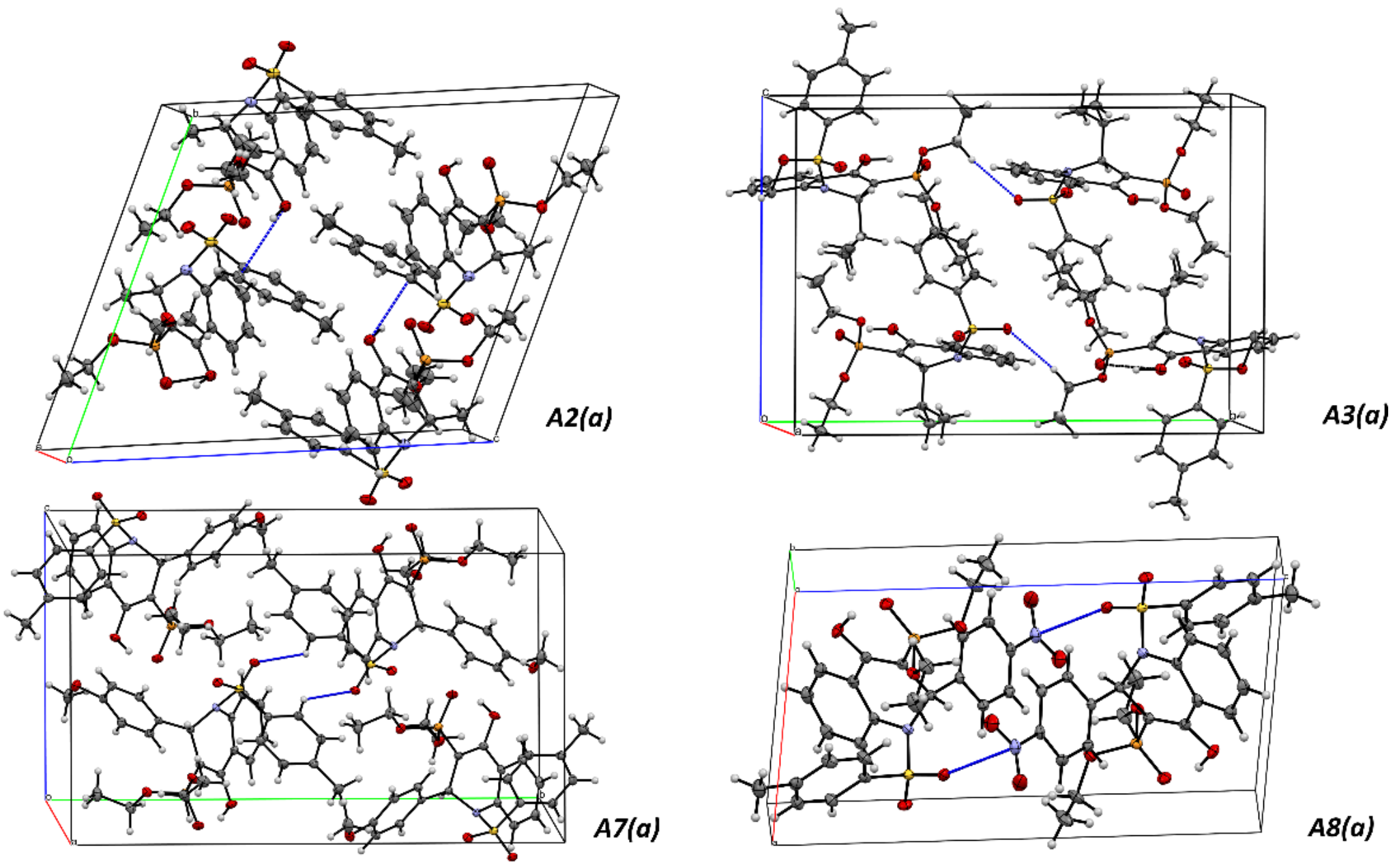

Figure S2. Partial packing diagrams of **A2(a)**, **A3(a)**, **A7(a)**, and **A8(a)**. Selected short (*vdW* radii - 0.1Å) intermolecular interactions (blue dotted lines) are shown.

Table S2. Bond lengths **for A2(a)**

Atom	Atom	Length/Å	Atom	Atom	Length/Å
P1B	O2B	1.4836(10)	C8A	C3A	1.404(2)
P1B	O4B	1.5783(10)	C8A	C7A	1.3966(19)
P1B	O3B	1.5737(10)	C1A	C2A	1.5216(18)
P1B	C1B	1.7590(13)	C1B	C2B	1.5221(18)
S1B	O8B	1.4307(10)	C3A	C4A	1.3916(19)
S1B	O7B	1.4315(10)	C3B	C4B	1.3897(19)
S1B	N1B	1.6636(11)	C7B	C6B	1.388(2)
S1B	C11B	1.7615(14)	C11B	C12B	1.3884(19)
P1A	O2A	1.4809(10)	C11B	C16B	1.3886(19)
P1A	O3A	1.5725(10)	C13B	C12B	1.382(2)
P1A	O4A	1.5752(10)	C13B	C14B	1.394(2)
P1A	C1A	1.7593(13)	C16A	C11A	1.395(2)
S1A	O8A	1.4312(11)	C16A	C15A	1.386(2)
S1A	O7A	1.4311(12)	C11A	C12A	1.3855(19)
S1A	N1A	1.6718(11)	C14B	C15B	1.391(2)
S1A	C11A	1.7588(14)	C14B	C142	1.506(2)
O1B	C9B	1.3462(16)	C2A	C21A	1.5309(18)
O1A	C9A	1.3461(16)	C5B	C4B	1.388(2)
O4B	C41B	1.4561(17)	C5B	C6B	1.388(2)
O3A	C31A	1.4597(16)	C12A	C13A	1.384(2)
O3B	C31B	1.473(6)	C7A	C6A	1.385(2)
O3B	C31C	1.463(6)	C15B	C16B	1.388(2)
O4A	C41A	1.4587(17)	C14A	C13A	1.391(2)
N1A	C3A	1.4375(18)	C14A	C15A	1.392(2)
N1A	C2A	1.4886(17)	C14A	C141	1.506(2)
N1B	C3B	1.4383(17)	C2B	C21B	1.5281(19)
N1B	C2B	1.4883(17)	C4A	C5A	1.387(2)
C9B	C8B	1.4686(18)	C41B	C42B	1.502(2)
C9B	C1B	1.3541(18)	C5A	C6A	1.386(2)
C8B	C3B	1.4030(19)	C41A	C42A	1.500(2)

Atom	Atom	Length/Å	Atom	Atom	Length/Å
C8B	C7B	1.3979(19)	C31A	C32A	1.498(2)
C9A	C8A	1.4685(18)	C31B	C32B	1.396(10)
C9A	C1A	1.3539(18)	C31C	C32C	1.433(8)

Table S3. Bond angles for **A2(a)**

Atom	Atom	Atom	Angle/˚	Atom	Atom	Atom	Angle/˚
O2B	P1B	O4B	113.78(6)	C2A	C1A	P1A	121.04(10)
O2B	P1B	O3B	115.98(6)	C9B	C1B	P1B	120.69(10)
O2B	P1B	C1B	112.04(6)	C9B	C1B	C2B	118.65(11)
O4B	P1B	C1B	109.27(6)	C2B	C1B	P1B	120.64(9)
O3B	P1B	O4B	101.46(6)	C8A	C3A	N1A	119.01(12)
O3B	P1B	C1B	103.31(6)	C4A	C3A	N1A	120.72(13)
O8B	S1B	O7B	119.85(6)	C4A	C3A	C8A	120.22(13)
O8B	S1B	N1B	106.60(6)	C8B	C3B	N1B	118.83(11)
O8B	S1B	C11B	107.49(7)	C4B	C3B	N1B	120.69(12)
O7B	S1B	N1B	106.15(6)	C4B	C3B	C8B	120.48(12)
O7B	S1B	C11B	108.51(6)	C6B	C7B	C8B	120.05(13)
N1B	S1B	C11B	107.71(6)	C12B	C11B	S1B	119.63(11)
O2A	P1A	O3A	115.47(6)	C12B	C11B	C16B	120.78(13)
O2A	P1A	O4A	114.19(6)	C16B	C11B	S1B	119.59(11)
O2A	P1A	C1A	111.64(6)	C12B	C13B	C14B	121.40(14)
O3A	P1A	O4A	101.15(6)	C15A	C16A	C11A	118.97(13)
O3A	P1A	C1A	104.22(6)	C13B	C12B	C11B	119.19(13)
O4A	P1A	C1A	109.25(6)	C16A	C11A	S1A	120.13(11)
O8A	S1A	N1A	106.45(6)	C12A	C11A	S1A	118.97(11)
O8A	S1A	C11A	107.49(7)	C12A	C11A	C16A	120.90(13)
O7A	S1A	O8A	119.97(7)	C13B	C14B	C142	120.34(15)
O7A	S1A	N1A	106.15(6)	C15B	C14B	C13B	118.23(14)
O7A	S1A	C11A	108.72(7)	C15B	C14B	C142	121.42(15)
N1A	S1A	C11A	107.47(6)	N1A	C2A	C1A	111.15(11)
C41B	O4B	P1B	122.45(9)	N1A	C2A	C21A	108.67(11)
C31A	O3A	P1A	119.06(9)	C1A	C2A	C21A	112.06(11)
C31B	O3B	P1B	120.1(3)	C4B	C5B	C6B	120.50(13)
C31C	O3B	P1B	118.8(3)	C13A	C12A	C11A	119.04(13)
C41A	O4A	P1A	122.31(9)	C5B	C4B	C3B	119.56(13)
C3A	N1A	S1A	114.15(9)	C6A	C7A	C8A	120.38(14)
C3A	N1A	C2A	114.49(11)	C16B	C15B	C14B	121.39(14)
C2A	N1A	S1A	117.65(9)	C13A	C14A	C15A	118.67(13)
C3B	N1B	S1B	115.45(9)	C13A	C14A	C141	120.51(15)
C3B	N1B	C2B	114.39(10)	C15A	C14A	C141	120.80(15)
C2B	N1B	S1B	118.33(9)	N1B	C2B	C1B	110.87(10)
O1B	C9B	C8B	114.13(11)	N1B	C2B	C21B	108.50(11)
O1B	C9B	C1B	125.58(12)	C1B	C2B	C21B	112.22(11)
C1B	C9B	C8B	120.29(12)	C12A	C13A	C14A	121.32(13)
C3B	C8B	C9B	119.22(12)	C7B	C6B	C5B	120.17(13)
C7B	C8B	C9B	121.55(12)	C16A	C15A	C14A	121.03(14)
C7B	C8B	C3B	119.21(12)	C15B	C16B	C11B	118.99(14)
O1A	C9A	C8A	113.88(11)	C5A	C4A	C3A	119.49(14)
O1A	C9A	C1A	125.49(12)	O4B	C41B	C42B	108.35(12)
C1A	C9A	C8A	120.61(12)	C6A	C5A	C4A	120.81(14)
C3A	C8A	C9A	119.13(12)	O4A	C41A	C42A	108.39(12)
C7A	C8A	C9A	121.63(13)	C7A	C6A	C5A	119.84(14)
C7A	C8A	C3A	119.20(12)	O3A	C31A	C32A	108.52(12)
C9A	C1A	P1A	120.48(10)	C32B	C31B	O3B	114.2(8)
C9A	C1A	C2A	118.45(12)	C32C	C31C	O3B	108.4(7)

Table S3. Torsion angles for **A2(a)**

A	B	C	D	Angle/˚	A	B	C	D	Angle/˚
P1B	O4B	C41B	C42B	-117.18(12)	N1B	S1B	C11B	C12B	99.01(11)
P1B	O3B	C31B	C32B	-176.5(5)	N1B	S1B	C11B	C16B	-80.64(12)
P1B	O3B	C31C	C32C	-177.1(4)	N1B	C3B	C4B	C5B	-177.40(12)
P1B	C1B	C2B	N1B	-147.29(9)	C9B	C8B	C3B	N1B	0.27(18)
P1B	C1B	C2B	C21B	91.20(13)	C9B	C8B	C3B	C4B	-178.96(12)
S1B	N1B	C3B	C8B	-109.92(12)	C9B	C8B	C7B	C6B	177.20(13)
S1B	N1B	C3B	C4B	69.31(15)	C9B	C1B	C2B	N1B	34.35(16)
S1B	N1B	C2B	C1B	92.83(12)	C9B	C1B	C2B	C21B	-87.16(15)
S1B	N1B	C2B	C21B	-143.50(10)	C8B	C9B	C1B	P1B	178.59(9)
S1B	C11B	C12B	C13B	179.04(10)	C8B	C9B	C1B	C2B	-3.05(18)
S1B	C11B	C16B	C15B	-179.65(11)	C8B	C3B	C4B	C5B	1.8(2)
P1A	O3A	C31A	C32A	-140.15(11)	C8B	C7B	C6B	C5B	1.6(2)
P1A	O4A	C41A	C42A	-113.40(13)	C9A	C8A	C3A	N1A	-0.09(18)
P1A	C1A	C2A	N1A	-147.59(9)	C9A	C8A	C3A	C4A	-177.59(12)
P1A	C1A	C2A	C21A	90.61(13)	C9A	C8A	C7A	C6A	175.62(12)
S1A	N1A	C3A	C8A	-107.82(12)	C9A	C1A	C2A	N1A	34.56(16)
S1A	N1A	C3A	C4A	69.67(14)	C9A	C1A	C2A	C21A	-87.25(15)
S1A	N1A	C2A	C1A	90.41(12)	C8A	C9A	C1A	P1A	178.01(9)
S1A	N1A	C2A	C21A	-145.83(10)	C8A	C9A	C1A	C2A	-4.13(18)
S1A	C11A	C12A	C13A	176.91(11)	C8A	C3A	C4A	C5A	2.2(2)
O1B	C9B	C8B	C3B	163.56(11)	C8A	C7A	C6A	C5A	1.6(2)
O1B	C9B	C8B	C7B	-14.93(18)	C1A	P1A	O3A	C31A	-176.93(11)
O1B	C9B	C1B	P1B	-0.65(19)	C1A	P1A	O4A	C41A	-97.65(11)
O1B	C9B	C1B	C2B	177.71(12)	C1A	C9A	C8A	C3A	-14.53(18)
O2B	P1B	O4B	C41B	24.86(12)	C1A	C9A	C8A	C7A	168.03(12)
O2B	P1B	O3B	C31B	44.8(6)	C1B	P1B	O4B	C41B	-101.19(11)
O2B	P1B	O3B	C31C	67.8(4)	C1B	P1B	O3B	C31B	167.8(6)
O2B	P1B	C1B	C9B	9.31(13)	C1B	P1B	O3B	C31C	-169.2(4)
O2B	P1B	C1B	C2B	-169.02(10)	C1B	C9B	C8B	C3B	-15.76(19)
O1A	C9A	C8A	C3A	164.03(11)	C1B	C9B	C8B	C7B	165.74(13)
O1A	C9A	C8A	C7A	-13.41(18)	C3A	N1A	C2A	C1A	-47.85(14)
O1A	C9A	C1A	P1A	-0.38(18)	C3A	N1A	C2A	C21A	75.91(14)
O1A	C9A	C1A	C2A	177.49(12)	C3A	C8A	C7A	C6A	-1.8(2)
O2A	P1A	O3A	C31A	60.25(12)	C3A	C4A	C5A	C6A	-2.5(2)
O2A	P1A	O4A	C41A	28.15(13)	C3B	N1B	C2B	C1B	-48.47(14)
O2A	P1A	C1A	C9A	10.62(13)	C3B	N1B	C2B	C21B	75.20(14)
O2A	P1A	C1A	C2A	-167.19(10)	C3B	C8B	C7B	C6B	-1.3(2)
O4B	P1B	O3B	C31B	-79.0(6)	C7B	C8B	C3B	N1B	178.80(12)
O4B	P1B	O3B	C31C	-56.0(4)	C7B	C8B	C3B	C4B	-0.4(2)
O4B	P1B	C1B	C9B	136.35(11)	C11B	S1B	N1B	C3B	58.29(11)
O4B	P1B	C1B	C2B	-41.97(12)	C11B	S1B	N1B	C2B	-82.61(11)
O3A	P1A	O4A	C41A	152.83(11)	C13B	C14B	C15B	C16B	-1.5(2)
O3A	P1A	C1A	C9A	-114.68(11)	C16A	C11A	C12A	C13A	-2.4(2)
O3A	P1A	C1A	C2A	67.51(11)	C12B	C11B	C16B	C15B	0.7(2)
O3B	P1B	O4B	C41B	150.15(10)	C12B	C13B	C14B	C15B	0.9(2)
O3B	P1B	C1B	C9B	-116.25(11)	C12B	C13B	C14B	C142	-178.35(14)
O3B	P1B	C1B	C2B	65.43(11)	C11A	S1A	N1A	C3A	57.72(11)
O8B	S1B	N1B	C3B	-56.82(11)	C11A	S1A	N1A	C2A	-80.68(11)
O8B	S1B	N1B	C2B	162.27(10)	C11A	C16A	C15A	C14A	0.3(2)
O8B	S1B	C11B	C12B	-146.47(11)	C11A	C12A	C13A	C14A	0.4(2)
O8B	S1B	C11B	C16B	33.88(13)	C14B	C13B	C12B	C11B	0.5(2)
O4A	P1A	O3A	C31A	-63.56(12)	C14B	C15B	C16B	C11B	0.7(2)
O4A	P1A	C1A	C9A	137.87(11)	C2A	N1A	C3A	C8A	31.92(16)
O4A	P1A	C1A	C2A	-39.94(12)	C2A	N1A	C3A	C4A	-150.59(12)
O7B	S1B	N1B	C3B	174.35(9)	C4B	C5B	C6B	C7B	-0.2(2)

A	B	C	D	Angle/˚	A	B	C	D	Angle/˚
O7B	S1B	N1B	C2B	33.44(11)	C7A	C8A	C3A	N1A	177.41(12)
O7B	S1B	C11B	C12B	-15.49(13)	C7A	C8A	C3A	C4A	-0.09(19)
O7B	S1B	C11B	C16B	164.86(11)	C2B	N1B	C3B	C8B	32.53(16)
O8A	S1A	N1A	C3A	-57.20(11)	C2B	N1B	C3B	C4B	-148.24(12)
O8A	S1A	N1A	C2A	164.39(10)	C13A	C14A	C15A	C16A	-2.2(2)
O8A	S1A	C11A	C16A	33.91(13)	C6B	C5B	C4B	C3B	-1.5(2)
O8A	S1A	C11A	C12A	-145.39(11)	C15A	C16A	C11A	S1A	-177.23(11)
O7A	S1A	N1A	C3A	173.91(9)	C15A	C16A	C11A	C12A	2.1(2)
O7A	S1A	N1A	C2A	35.51(11)	C15A	C14A	C13A	C12A	1.9(2)
O7A	S1A	C11A	C16A	165.19(11)	C16B	C11B	C12B	C13B	-1.3(2)
O7A	S1A	C11A	C12A	-14.11(13)	C4A	C5A	C6A	C7A	0.6(2)
N1A	S1A	C11A	C16A	-80.32(12)	C142	C14B	C15B	C16B	177.71(15)
N1A	S1A	C11A	C12A	100.38(11)	C141	C14A	C13A	C12A	-176.68(14)
N1A	C3A	C4A	C5A	-175.23(12)	C141	C14A	C15A	C16A	176.35(14)

Table S4. Bond lengths for **A3(a)**

Atom	Atom	Length/Å	Atom	Atom	Length/Å
S1	O7	1.4308(10)	C8	C7	1.3978(18)
S1	O8	1.4333(10)	C14	C13	1.3902(19)
S1	N1	1.6617(11)	C14	C141	1.5014(19)
S1	C11	1.7602(13)	C14	C15	1.394(2)
P1	O3	1.5713(10)	C3	C4	1.3905(19)
P1	O4	1.5772(9)	C2	C21	1.5475(18)
P1	O2	1.4829(10)	C12	C11	1.3868(19)
P1	C1	1.7638(13)	C12	C13	1.3889(19)
O3	C31	1.4655(16)	C11	C16	1.3908(19)
O1	C9	1.3473(15)	C7	C6	1.384(2)
O4	C41	1.4602(17)	C21	C23	1.525(2)
N1	C3	1.4367(16)	C21	C22	1.5298(18)
N1	C2	1.4893(15)	C6	C5	1.387(2)
C9	C1	1.3565(18)	C4	C5	1.391(2)
C9	C8	1.4665(18)	C31	C32	1.491(2)
C1	C2	1.5215(17)	C16	C15	1.381(2)
C8	C3	1.4014(18)	C41	C42	1.492(2)

Table S5. Bond angles for **A3(a)**

Atom	Atom	Atom	Angle/˚	Atom	Atom	Atom	Angle/˚
O7	S1	O8	119.61(6)	C13	C14	C141	121.98(12)
O7	S1	N1	106.47(6)	C13	C14	C15	118.56(13)
O7	S1	C11	108.56(6)	C15	C14	C141	119.46(12)
O8	S1	N1	107.02(6)	C8	C3	N1	119.02(12)
O8	S1	C11	107.66(6)	C4	C3	N1	120.96(12)
N1	S1	C11	106.88(6)	C4	C3	C8	120.01(12)
O3	P1	O4	101.04(5)	N1	C2	C1	111.39(10)
O3	P1	C1	104.25(5)	N1	C2	C21	110.20(10)
O4	P1	C1	110.24(6)	C1	C2	C21	115.17(11)
O2	P1	O3	116.12(6)	C11	C12	C13	118.93(12)
O2	P1	O4	113.52(5)	C12	C11	S1	120.08(10)
O2	P1	C1	110.91(6)	C12	C11	C16	120.78(12)
C31	O3	P1	120.94(9)	C16	C11	S1	119.11(10)
C41	O4	P1	119.91(9)	C12	C13	C14	121.26(12)
C3	N1	S1	116.71(9)	C6	C7	C8	120.18(13)
C3	N1	C2	116.78(10)	C23	C21	C2	114.46(11)
C2	N1	S1	116.60(8)	C23	C21	C22	110.89(12)
O1	C9	C1	125.41(12)	C22	C21	C2	110.05(11)
O1	C9	C8	113.72(11)	C7	C6	C5	120.07(13)

Atom	Atom	Atom	Angle/˚	Atom	Atom	Atom	Angle/˚
C1	C9	C8	120.87(11)	C3	C4	C5	119.73(13)
C9	C1	P1	119.41(10)	O3	C31	C32	110.90(12)
C9	C1	C2	119.99(11)	C6	C5	C4	120.46(13)
C2	C1	P1	120.55(9)	C15	C16	C11	119.36(13)
C3	C8	C9	119.04(12)	O4	C41	C42	108.34(12)
C7	C8	C9	121.43(12)	C16	C15	C14	121.02(13)
C7	C8	C3	119.51(12)				

Table S6. Torsion angles for **A3(a)**

A	B	C	D	Angle/˚	A	B	C	D	Angle/˚
S1	N1	C3	C8	-113.95(12)	C9	C1	C2	N1	26.11(16)
S1	N1	C3	C4	66.56(15)	C9	C1	C2	C21	-100.33(14)
S1	N1	C2	C1	103.11(11)	C9	C8	C3	N1	-1.73(18)
S1	N1	C2	C21	-127.77(10)	C9	C8	C3	C4	177.77(12)
S1	C11	C16	C15	-179.23(12)	C9	C8	C7	C6	-179.20(13)
P1	O3	C31	C32	-94.35(14)	C1	P1	O3	C31	-173.75(10)
P1	O4	C41	C42	177.46(10)	C1	P1	O4	C41	-84.50(11)
P1	C1	C2	N1	-151.43(9)	C1	C9	C8	C3	-14.59(19)
P1	C1	C2	C21	82.13(13)	C1	C9	C8	C7	164.48(13)
O3	P1	O4	C41	165.67(10)	C1	C2	C21	C23	60.31(15)
O3	P1	C1	C9	-115.91(11)	C1	C2	C21	C22	-174.03(11)
O3	P1	C1	C2	61.64(11)	C8	C9	C1	P1	178.58(10)
O7	S1	N1	C3	-179.77(9)	C8	C9	C1	C2	1.01(19)
O7	S1	N1	C2	35.53(10)	C8	C3	C4	C5	1.9(2)
O7	S1	C11	C12	-22.26(13)	C8	C7	C6	C5	1.0(2)
O7	S1	C11	C16	159.40(11)	C3	N1	C2	C1	-41.56(15)
O1	C9	C1	P1	-2.22(18)	C3	N1	C2	C21	87.56(13)
O1	C9	C1	C2	-179.79(12)	C3	C8	C7	C6	-0.1(2)
O1	C9	C8	C3	166.12(11)	C3	C4	C5	C6	-1.0(2)
O1	C9	C8	C7	-14.80(18)	C2	N1	C3	C8	30.68(16)
O4	P1	O3	C31	-59.34(10)	C2	N1	C3	C4	-148.81(13)
O4	P1	C1	C9	136.38(10)	C12	C11	C16	C15	2.4(2)
O4	P1	C1	C2	-46.07(11)	C11	S1	N1	C3	64.34(10)
O8	S1	N1	C3	-50.77(10)	C11	S1	N1	C2	-80.36(10)
O8	S1	N1	C2	164.53(9)	C11	C12	C13	C14	0.3(2)
O8	S1	C11	C12	-153.09(11)	C11	C16	C15	C14	0.0(2)
O8	S1	C11	C16	28.56(13)	C13	C14	C15	C16	-2.2(2)
O2	P1	O3	C31	63.93(11)	C13	C12	C11	S1	179.12(10)
O2	P1	O4	C41	40.62(11)	C13	C12	C11	C16	-2.6(2)
O2	P1	C1	C9	9.77(13)	C7	C8	C3	N1	179.17(12)
O2	P1	C1	C2	-172.67(9)	C7	C8	C3	C4	-1.3(2)
N1	S1	C11	C12	92.22(11)	C7	C6	C5	C4	-0.4(2)
N1	S1	C11	C16	-86.12(12)	C141	C14	C13	C12	-177.69(13)
N1	C3	C4	C5	-178.60(13)	C141	C14	C15	C16	177.56(15)
N1	C2	C21	C23	-66.73(14)	C15	C14	C13	C12	2.1(2)
N1	C2	C21	C22	58.93(14)					

Table S7. Bond lengths for **A7(a)**

Atom	Atom	Length/Å	Atom	Atom	Length/Å
S1	N1	1.6594(9)	C6	C7	1.3852(17)
S1	O7	1.4343(8)	C7	C8	1.3987(16)
S1	O8	1.4345(8)	C8	C9	1.4683(15)
S1	C11	1.7592(11)	O9	C27	1.4257(16)
O1	C9	1.3479(13)	O9	C24	1.3679(14)
N1	C3	1.4361(14)	C31	C32	1.5024(17)
N1	C2	1.5000(13)	C26	C25	1.3920(16)

Atom	Atom	Length/Å	Atom	Atom	Length/Å
C1	P1	1.7654(11)	C26	C21	1.3886(15)
C1	C2	1.5170(14)	C25	C24	1.3930(16)
C1	C9	1.3556(16)	C24	C23	1.3939(17)
P1	O3	1.5697(9)	C23	C22	1.3835(16)
P1	O2	1.4824(9)	C22	C21	1.3973(15)
P1	O4	1.5812(9)	C16	C15	1.3881(17)
O3	C31	1.4655(13)	C16	C11	1.3936(16)
C3	C4	1.3926(15)	C15	C14	1.3952(17)
C3	C8	1.4048(15)	C14	C13	1.3937(16)
C2	C21	1.5168(15)	C14	C141	1.5040(16)
O4	C41	1.4636(13)	C13	C12	1.3873(16)
C4	C5	1.3922(16)	C12	C11	1.3894(16)
C5	C6	1.3886(18)	C41	C42	1.5035(17)

Table S8. Bond angles for **A7(a)**

Atom	Atom	Atom	Angle/˚	Atom	Atom	Atom	Angle/˚
N1	S1	C11	106.44(5)	C3	C8	C9	119.12(10)
O7	S1	N1	105.51(5)	C7	C8	C3	119.45(10)
O7	S1	O8	119.40(5)	C7	C8	C9	121.41(10)
O7	S1	C11	109.29(5)	C24	O9	C27	116.73(10)
O8	S1	N1	107.28(5)	O1	C9	C1	125.42(10)
O8	S1	C11	108.18(5)	O1	C9	C8	113.97(10)
C3	N1	S1	117.16(7)	C1	C9	C8	120.61(10)
C3	N1	C2	116.08(8)	O3	C31	C32	108.10(10)
C2	N1	S1	117.11(7)	C21	C26	C25	121.29(11)
C2	C1	P1	120.10(8)	C26	C25	C24	119.40(11)
C9	C1	P1	119.87(8)	O9	C24	C25	124.60(11)
C9	C1	C2	120.01(10)	O9	C24	C23	115.55(10)
O3	P1	C1	104.28(5)	C25	C24	C23	119.85(10)
O3	P1	O4	101.35(5)	C22	C23	C24	120.03(11)
O2	P1	C1	110.49(5)	C23	C22	C21	120.82(11)
O2	P1	O3	116.87(5)	C26	C21	C2	122.70(10)
O2	P1	O4	113.53(5)	C26	C21	C22	118.56(10)
O4	P1	C1	109.56(5)	C22	C21	C2	118.64(10)
C31	O3	P1	120.44(7)	C15	C16	C11	118.93(11)
C4	C3	N1	120.96(10)	C16	C15	C14	121.45(11)
C4	C3	C8	119.93(10)	C15	C14	C141	121.61(11)
C8	C3	N1	119.11(10)	C13	C14	C15	118.22(11)
N1	C2	C1	111.31(9)	C13	C14	C141	120.17(11)
N1	C2	C21	108.13(8)	C12	C13	C14	121.44(11)
C21	C2	C1	115.70(9)	C13	C12	C11	119.11(11)
C41	O4	P1	116.60(7)	C16	C11	S1	119.31(9)
C5	C4	C3	119.70(11)	C12	C11	S1	119.72(9)
C6	C5	C4	120.63(11)	C12	C11	C16	120.84(10)
C7	C6	C5	119.86(11)	O4	C41	C42	108.34(10)
C6	C7	C8	120.37(11)				

Table S9. Torsion angles for **A7(a)**

A	B	C	D	Angle/˚	A	B	C	D	Angle/˚
S1	N1	C3	C4	-64.67(12)	C6	C7	C8	C9	-177.99(11)
S1	N1	C3	C8	114.77(10)	C7	C8	C9	O1	13.78(15)
S1	N1	C2	C1	-102.46(9)	C7	C8	C9	C1	-166.11(11)
S1	N1	C2	C21	129.40(8)	O7	S1	N1	C3	-179.12(8)
N1	S1	C11	C16	-79.50(10)	O7	S1	N1	C2	-34.25(9)
N1	S1	C11	C12	96.51(10)	O7	S1	C11	C16	34.00(10)
N1	C3	C4	C5	176.79(10)	O7	S1	C11	C12	-149.99(9)

A	B	C	D	Angle/˚	A	B	C	D	Angle/˚
N1	C3	C8	C7	-177.94(10)	C8	C3	C4	C5	-2.65(16)
N1	C3	C8	C9	0.71(15)	O8	S1	N1	C3	52.57(9)
N1	C2	C21	C26	87.55(12)	O8	S1	N1	C2	-162.56(7)
N1	C2	C21	C22	-88.78(11)	O8	S1	C11	C16	165.48(9)
C1	P1	O3	C31	-179.20(9)	O8	S1	C11	C12	-18.51(11)
C1	P1	O4	C41	77.25(9)	O9	C24	C23	C22	-177.84(10)
C1	C2	C21	C26	-38.04(14)	C9	C1	P1	O3	125.84(9)
C1	C2	C21	C22	145.62(10)	C9	C1	P1	O2	-0.53(11)
P1	C1	C2	N1	150.57(8)	C9	C1	P1	O4	-126.35(9)
P1	C1	C2	C21	-85.48(11)	C9	C1	C2	N1	-27.97(14)
P1	C1	C9	O1	1.48(16)	C9	C1	C2	C21	95.98(12)
P1	C1	C9	C8	-178.64(8)	C27	O9	C24	C25	-5.19(17)
P1	O3	C31	C32	163.32(9)	C27	O9	C24	C23	175.16(11)
P1	O4	C41	C42	-159.93(8)	C26	C25	C24	O9	178.39(11)
O3	P1	O4	C41	-172.97(8)	C26	C25	C24	C23	-1.98(17)
C3	N1	C2	C1	42.79(12)	C25	C26	C21	C2	-174.71(10)
C3	N1	C2	C21	-85.35(11)	C25	C26	C21	C22	1.63(16)
C3	C4	C5	C6	1.68(17)	C25	C24	C23	C22	2.49(17)
C3	C8	C9	O1	-164.84(10)	C24	C23	C22	C21	-0.94(17)
C3	C8	C9	C1	15.26(16)	C23	C22	C21	C2	175.38(10)
C2	N1	C3	C4	150.10(10)	C23	C22	C21	C26	-1.11(16)
C2	N1	C3	C8	-30.45(13)	C21	C26	C25	C24	-0.09(17)
C2	C1	P1	O3	-52.70(9)	C16	C15	C14	C13	0.31(18)
C2	C1	P1	O2	-179.08(8)	C16	C15	C14	C141	-179.75(11)
C2	C1	P1	O4	55.11(10)	C15	C16	C11	S1	175.31(9)
C2	C1	C9	O1	-179.98(10)	C15	C16	C11	C12	-0.65(17)
C2	C1	C9	C8	-0.10(16)	C15	C14	C13	C12	-1.33(18)
O2	P1	O3	C31	-56.93(10)	C14	C13	C12	C11	1.35(18)
O2	P1	O4	C41	-46.80(9)	C13	C12	C11	S1	-176.29(9)
O4	P1	O3	C31	67.00(9)	C13	C12	C11	C16	-0.33(17)
C4	C3	C8	C7	1.51(16)	C11	S1	N1	C3	-63.05(9)
C4	C3	C8	C9	-179.84(10)	C11	S1	N1	C2	81.81(8)
C4	C5	C6	C7	0.47(18)	C11	C16	C15	C14	0.67(18)
C5	C6	C7	C8	-1.62(18)	C141	C14	C13	C12	178.73(11)
C6	C7	C8	C3	0.64(17)					

Table S10. Bond lengths for **A8(a)**

Atom	Atom	Length/Å	Atom	Atom	Length/Å
S1	N1	1.6551(10)	C4	C5	1.3906(18)
S1	O6	1.4330(9)	C5	C6	1.386(2)
S1	O7	1.4313(9)	C6	C7	1.3846(19)
S1	C11	1.7576(13)	C7	C8	1.4016(17)
P1	C1	1.7629(12)	C8	C9	1.4654(17)
P1	O2	1.4828(9)	C141	C14	1.5045(18)
P1	O3	1.5678(9)	C11	C16	1.3899(18)
P1	O4	1.5736(9)	C11	C12	1.3908(18)
O1	C9	1.3463(15)	C31	C32	1.497(2)
N1	C2	1.4845(15)	C26	C25	1.3881(17)
N1	C3	1.4333(15)	C26	C21	1.3990(17)
C1	C2	1.5156(16)	C25	C24	1.3840(18)
C1	C9	1.3554(17)	C24	C23	1.3816(18)
C2	C21	1.5343(16)	C23	C22	1.3913(17)
N2	O8	1.2270(16)	C22	C21	1.3902(17)
N2	O9	1.2242(16)	C16	C15	1.3869(19)
N2	C24	1.4740(15)	C15	C14	1.3933(19)
C3	C4	1.3949(18)	C14	C13	1.3917(19)
C3	C8	1.4038(18)	C13	C12	1.3864(19)

Atom	Atom	Length/Å	Atom	Atom	Length/Å
O3	C31	1.4643(15)	C41	C42	1.503(2)
O4	C41	1.4594(15)			

Table S11. Bond angles for **A8(a)**

Atom	Atom	Atom	Angle/˚	Atom	Atom	Atom	Angle/˚
N1	S1	C11	106.87(5)	C7	C6	C5	119.85(12)
O6	S1	N1	105.93(5)	C6	C7	C8	120.42(12)
O6	S1	C11	109.29(6)	C3	C8	C9	119.22(11)
O7	S1	N1	107.61(5)	C7	C8	C3	119.27(12)
O7	S1	O6	119.27(6)	C7	C8	C9	121.41(12)
O7	S1	C11	107.25(6)	O1	C9	C1	125.01(11)
O2	P1	C1	111.01(6)	O1	C9	C8	114.39(11)
O2	P1	O3	116.00(5)	C1	C9	C8	120.56(11)
O2	P1	O4	114.37(5)	C16	C11	S1	119.45(10)
O3	P1	C1	103.02(5)	C16	C11	C12	121.06(12)
O3	P1	O4	102.49(5)	C12	C11	S1	119.49(10)
O4	P1	C1	109.01(5)	O3	C31	C32	107.54(11)
C2	N1	S1	119.76(8)	C25	C26	C21	120.85(12)
C3	N1	S1	119.17(8)	C24	C25	C26	118.10(12)
C3	N1	C2	116.11(10)	C25	C24	N2	118.89(11)
C2	C1	P1	121.52(9)	C23	C24	N2	118.29(12)
C9	C1	P1	120.04(9)	C23	C24	C25	122.77(11)
C9	C1	C2	118.39(11)	C24	C23	C22	118.17(12)
N1	C2	C1	110.74(9)	C21	C22	C23	120.89(12)
N1	C2	C21	107.82(9)	C26	C21	C2	118.55(11)
C1	C2	C21	115.13(10)	C22	C21	C2	122.28(11)
O8	N2	C24	117.94(11)	C22	C21	C26	119.15(11)
O9	N2	O8	124.13(11)	C15	C16	C11	118.91(12)
O9	N2	C24	117.92(11)	C16	C15	C14	121.32(12)
C4	C3	N1	121.71(11)	C15	C14	C141	121.48(13)
C4	C3	C8	120.04(11)	C13	C14	C141	120.06(13)
C8	C3	N1	118.25(11)	C13	C14	C15	118.43(12)
C31	O3	P1	120.64(8)	C12	C13	C14	121.40(12)
C41	O4	P1	122.56(9)	C13	C12	C11	118.86(12)
C5	C4	C3	119.59(12)	O4	C41	C42	109.44(12)
C6	C5	C4	120.79(12)				

Table S12. Torsion angles for **A8(a)**

A	B	C	D	Angle/˚	A	B	C	D	Angle/˚
S1	N1	C2	C1	-108.17(10)	C4	C3	C8	C7	-1.28(18)
S1	N1	C2	C21	125.06(9)	C4	C3	C8	C9	175.02(11)
S1	N1	C3	C4	-52.85(14)	C4	C5	C6	C7	-1.3(2)
S1	N1	C3	C8	126.82(10)	C5	C6	C7	C8	-0.02(19)
S1	C11	C16	C15	-179.07(10)	C6	C7	C8	C3	1.32(18)
S1	C11	C12	C13	179.08(10)	C6	C7	C8	C9	-174.90(11)
P1	C1	C2	N1	142.62(9)	O6	S1	N1	C2	-20.31(10)
P1	C1	C2	C21	-94.76(11)	O6	S1	N1	C3	-174.47(9)
P1	C1	C9	O1	4.17(17)	O6	S1	C11	C16	36.12(12)
P1	C1	C9	C8	-173.46(9)	O6	S1	C11	C12	-143.93(10)
P1	O3	C31	C32	170.01(9)	C7	C8	C9	O1	15.80(17)
P1	O4	C41	C42	102.12(13)	C7	C8	C9	C1	-166.33(12)
N1	S1	C11	C16	-78.09(11)	O7	S1	N1	C2	-148.92(9)
N1	S1	C11	C12	101.86(11)	O7	S1	N1	C3	56.92(10)
N1	C2	C21	C26	-48.19(14)	O7	S1	C11	C16	166.75(10)
N1	C2	C21	C22	133.12(11)	O7	S1	C11	C12	-13.30(12)
N1	C3	C4	C5	179.62(11)	C8	C3	C4	C5	-0.05(18)

A	B	C	D	Angle/°	A	B	C	D	Angle/°
N1	C3	C8	C7	179.04(11)	O8	N2	C24	C25	-12.33(17)
N1	C3	C8	C9	-4.66(16)	O8	N2	C24	C23	170.36(12)
C1	P1	O3	C31	173.21(10)	C9	C1	C2	N1	-34.59(14)
C1	P1	O4	C41	101.17(10)	C9	C1	C2	C21	88.03(13)
C1	C2	C21	C26	-172.35(11)	O9	N2	C24	C25	166.80(12)
C1	C2	C21	C22	8.95(16)	O9	N2	C24	C23	-10.50(17)
C2	N1	C3	C4	152.07(11)	C141	C14	C13	C12	-177.28(13)
C2	N1	C3	C8	-28.25(15)	C11	S1	N1	C2	96.15(9)
C2	C1	C9	O1	-178.57(11)	C11	S1	N1	C3	-58.00(10)
C2	C1	C9	C8	3.80(17)	C11	C16	C15	C14	0.0(2)
O2	P1	C1	C2	167.52(9)	C26	C25	C24	N2	-175.15(11)
O2	P1	C1	C9	-15.32(12)	C26	C25	C24	C23	2.03(19)
O2	P1	O3	C31	-65.31(11)	C25	C26	C21	C2	179.09(11)
O2	P1	O4	C41	-23.75(11)	C25	C26	C21	C22	-2.18(18)
N2	C24	C23	C22	174.90(11)	C25	C24	C23	C22	-2.30(19)
C3	N1	C2	C1	46.75(13)	C24	C23	C22	C21	0.27(19)
C3	N1	C2	C21	-80.02(12)	C23	C22	C21	C2	-179.41(11)
C3	C4	C5	C6	1.37(19)	C23	C22	C21	C26	1.90(18)
C3	C8	C9	O1	-160.42(11)	C21	C26	C25	C24	0.26(18)
C3	C8	C9	C1	17.45(17)	C16	C11	C12	C13	-0.96(19)
O3	P1	C1	C2	-67.68(10)	C16	C15	C14	C141	177.27(13)
O3	P1	C1	C9	109.49(10)	C16	C15	C14	C13	-1.0(2)
O3	P1	O4	C41	-150.14(9)	C15	C14	C13	C12	1.0(2)
O4	P1	C1	C2	40.66(11)	C14	C13	C12	C11	0.0(2)
O4	P1	C1	C9	-142.18(10)	C12	C11	C16	C15	1.0(2)
O4	P1	O3	C31	60.02(10)					

Table S13. Geometrical Parameters of selected intramolecular hydrogen bonds in **A2(a)**, **A3(a)**, **A7(a)**, and **A8(a)**

	D–H···A	d(D–H) [Å]	d(H···A) [Å]	d(D···A) [Å]	D–H···A [°]
**A2(a)**	O1A–H1A···O2A	1.01(2)	1.714(19)	2.668(2)	156(2)
	O1B–H1B···O2B	1.01(2)	1.72(2)	2.684(2)	157(2)
	C2A–H2A···O7A	1.00	2.36	2.881(2)	112
	C2B–H2B···O7B	1.00	2.35	2.874(2)	112
	C4A–H4A···O8A	0.95	2.55	2.927(2)	104
	C4B–H4B···O8B	0.95	2.54	2.951(2)	106
	C12A–H12A···O7A	0.95	2.54	2.910 (2)	103
	C12B–H12B···O7B	0.95	2.56	2.927(2)	103
	C41B–H41B···O2B	0.99	2.56	3.013(2)	108
**A3(a)**	O1–H1···O2	1.012(5)	1.645(9)	2.6089(14)	157.4(14)
	C2–H2···O7	1.00	2.29	2.8562(16)	115
	C4–H4···O8	0.95	2.49	2.9158(18)	107
	C31–H31B···O4	0.99	2.56	2.9818(17)	106
**A7(a)**	O1–H1···O2	1.012(17)	1.638(17)	2.6010(14)	157.3(17)
	C2–H2···O7	1.00	2.28	2.8420(14)	114
	C4–H4···O8	0.95	2.49	2.9262(16)	108
	C12–H12···O8	0.95	2.56	2.9289(16)	103
	C16–H16···O3	0.95	2.49	3.2390(16)	136
**A8(a)**	O1–H1···O2	1.012(15)	1.675(17)	2.6449(13)	159.1(17)
	C2–H2···O6	1.00	2.26	2.8261(17)	114
	C4–H4···O7	0.95	2.35	2.9360(18)	120
	C12–H12···O7	0.95	2.52	2.8864(16)	103
	C16–H16···O3	0.95	2.48	3.3079(18)	146

Table S14. Geometrical Parameters of selected intermolecular hydrogen bonds in **A2(a)**, **A3(a)**, **A7(a)**, and **A8(a)**

	D–H···A	d(D–H) [Å]	d(H···A) [Å]	d(D···A) [Å]	D–H···A [°]
**A2(a)**	C41B–H41A···O7B^{I}	0.99	2.47	3.2582(19)	136
	C41A–H41C···O7A^{II}	0.99	2.56	3.3408(18)	135
	C5–H5···O2^{III}	0.95	2.48	3.2184(18)	135

**A3(a)**	C7–H7…O^{IV}	0.95	2.57	3.4321(17)	152
	C31–H31B···O8^{V}	0.99	2.55	3.4762(17)	156
**A7(a)**	C12–H12···O8^{VI}	0.95	2.53	3.2253(16)	130
	C41–H41B···O7^{VII}	0.99	2.39	3.2736(16)	148
**A8(a)**	C31–H31A···O7^{VIII}	0.99	2.44	3.1837(17)	131
	C41–H41B···O9^{IX}	0.99	2.48	3.179(2)	127

Symmetry: $^{I)}$*1−x, −y, 2−z;*$^{II)}$*−x, 1−y, 2−z;*$^{III)}$*½−x, ½+y, ½−z;* $^{IV)}$*3/2−x, −½+y, ½−z;*$^{V)}$*−1+x, y, z;* $^{VI)}$*1−x, 1−y, 1−z;* $^{VII)}$*−½+x, ½−y, −½+z;*$^{VIII)}$ *−1+x, −1+y, z;*$^{IX)}$ *−x, 1−y, 1−z*

S2. Neutron Diffraction Analysis

Neutron diffraction data were collected for the selected crystal to localize the hydrogen atom involved in hydrogen bonding**.** Obtained distances served as reference values for DFIX and DANG restraints for O1-H1 bond for good-quality SCXRD structures of **A2(a)**, **A3(a)**, **A7(a)**, and **A8(a).**

a) General comments

A crystal acceptable for single-crystal neutron diffraction was obtained using the slow evaporation method. The compound crystallized from a saturated acetonitrile solution. The crystallization system was kept in the fridge for approximately 2 weeks. Neutron diffraction data on a crystal of **A7(a)$_n$** (size 2.2 x 2 x 1 mm) were collected at 100(2) K on the SXD single-crystal diffractometer of the ISIS Neutron and Muon Source (UK)[5], using the Laue method in Time-of-Flight. The SXD instrument, which receives neutrons from the water moderator at RT, is equipped with an array of eleven 2D position-sensitive detectors statically placed around the sample position, providing a large coverage of reciprocal space, and uses polychromatic radiation in the range 0.3-6 Å. Data were collected in a series of 5 discrete orientations of the sample around the instrument's vertical axis, with a counting time of ~5 h 30m for each orientation. Data were initially indexed using the unit cell from the X-ray measurement, and integrated intensities were extracted using the 3D-profile fitting method implemented in the SXD2001 software[6], corrected for the Lorentz effect. No additional wavelength-dependent corrections were applied to the data. Final unit-cell parameters were refined against the fitted positions of the Bragg diffraction peaks, after the 3D profile integration.

b) Structure solution and refinement

Due to the limited crystal size (approximately 2.2 x 2 x 1 mm), close to the practical limits of the neutron diffraction method, the overall data quality was insufficient for a fully unconstrained structural refinement; therefore, isotropic displacement parameters were applied for the phosphorus atom, and selected displacement parameters for carbon and oxygen atoms were constrained (EADP). The hydrogen atom position obtained from the neutron experiment was subsequently used to define the hydrogen-bonding geometry in the high-quality SCXRD structure used to discuss structural features.
The structural refinement of **A7(a)$_n$** at 100K was performed using a starting model from a previous X-ray measurement (see A7(a)); the reflection conditions were consistent with the previously reported $P2_1/n$ space group. A total of 9233 reflections were used in the refinement, with d_{min} ~ 0.33 Å ($-20 \le h \le +19$, $-57 \le k \le +44$ and $-23 \le l \le +15$;), all with $I_o > 2\sigma(I_o)$. The refinement was conducted using the SHELXL-2019/3 software[3], by full matrix least squares on F^2. All hydrogen atoms were located by Fourier difference map and freely refined with independent anisotropic displacement parameters. No secondary isotropic extinction parameter was refined.

File CCDC 2547090 contains supplementary crystallographic data for this paper. This data can be obtained free of charge from the Cambridge Crystallographic Data Centre via www.ccdc.cam.ac.uk/structures.

Table S15. Neutron diffraction data collection and refinement parameters.

**Parameter**	**A7(a)$_n$** **CCDC: 2547090**
Instrument / Beamline	SXD/ISIS
Wavelength	White-beam (0.3 – 6.0 Å)
Temperature / K	100(2)
Crystal size / mm	2.2 × 1.8 × 1.0
Space group	$P2_1/c$
a / Å	10.7619(2)
b / Å	20.7037(3)
c / Å	12.05970(3)
α / °	90
β / °	90.52
γ / °	90
V / Å^3	2650.04(6)
Refl. collected	9233
R_{int}	0.36
$R1$, w$R2$ [$I > 2\sigma(I)$]	0.1245, 0.3127
GOF on F^2	1.066

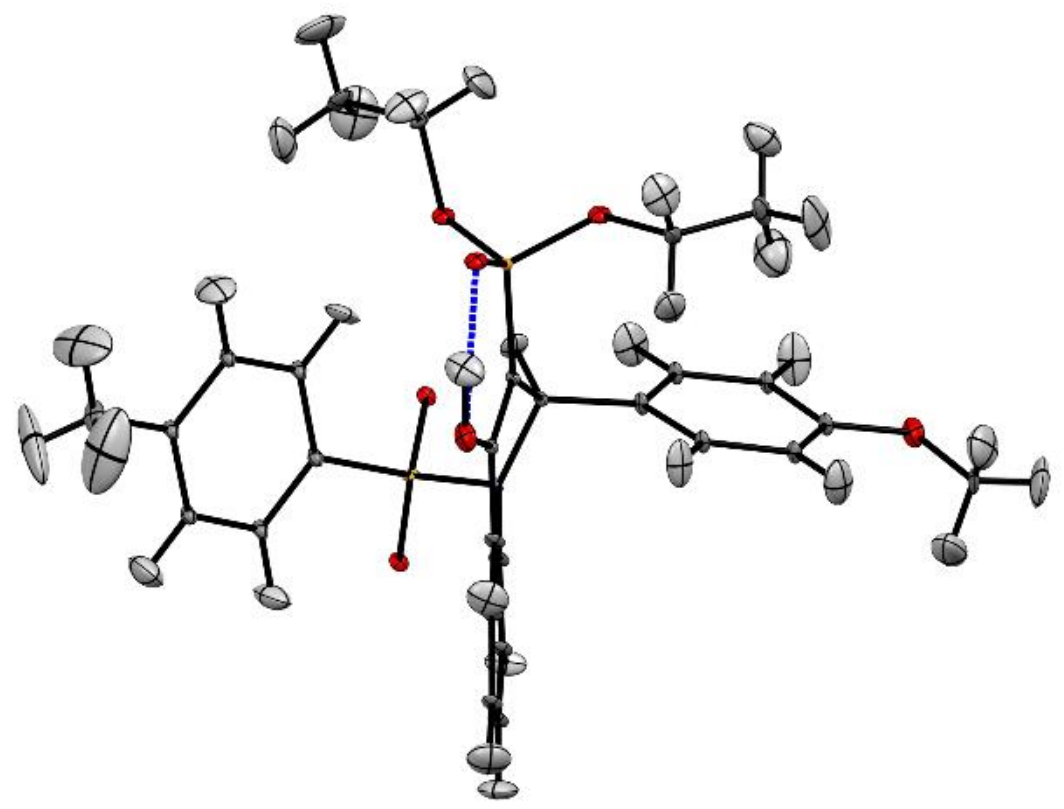

Figure S3. Molecular structure of **A7(a)$_n$** derived from a single-crystal neutron diffraction experiment. Atomic displacement ellipsoids are drawn at 50% probability level. All H atoms are shown explicitly.

S3. HOMA Analysis

Aromaticity indices for O-C-C-P-O regions of the *quasi*-ring in studied structures were quantified using the Harmonic Oscillator Model of Aromaticity (HOMA)[7] according to the expression:

$$\mathrm{HOMA} = 1 - \frac{\alpha}{n}\sum_{i=1}^{n}(R_i - R_{\mathrm{opt}})^2$$

where n is the number of bonds included in the aromatic circuit, R_i are the individual bond lengths extracted from SCXRD geometries, R_{opt} is the optimal bond length corresponding to complete π-electron delocalization for a given bond type, and α is an empirical normalization constant.

For C–C, C–O, and C–P bonds, previously reported HOMA parameters were used[8]. Since no α and R_{opt} parameters have been reported in the literature for P–O bonds, these quantities were determined explicitly for the present study.

Determination of R_{opt} and α for P–O bond

The optimal bond length R_{opt} is the length of a bond for which equal energy inputs are required to extend it to the length of the single bond or to compress it to the length of the double bond.:

$$R_{\mathrm{opt}}(\mathrm{P-O}) = \frac{R(\mathrm{P-O})_{\mathrm{s}} + 2R(\mathrm{P=O})_{\mathrm{d}}}{3}$$

The reference P–O single and double bond lengths (R_s = 1.64 Å and R_d = 1.51 Å) were adopted from the detailed electronic-structure analysis of phosphorus–oxygen bonding reported by Gamoke, Neff, and Simons.[9]These values correspond to well-defined localized P–O and P=O bonding situations in phosphates and phosphine oxides, and therefore fulfill the requirements of non-delocalized reference systems within the HOMA formalism.[9] Based on these values, R_{opt}(P–O) was determined to be 1.560 Å. The normalization constant α was then calculated using the condition that the mean-square deviation of the single and double bond reference lengths from R_{opt} yields HOMA = 0 for a fully localized reference system:

$$\alpha(\mathrm{P-O}) = \frac{2}{(R_{\mathrm{s}} - R_{\mathrm{opt}})^2 + (R_{\mathrm{d}} - R_{\mathrm{opt}})^2}$$

This procedure ensures consistency with the original HOMA definition and allows the P–O bond to be treated on the same footing as other heteroatomic bonds in aromaticity assessments. The α and R_{opt} parameters used in this work are summarized in Table S1.

Table S14. Parameters used for the evaluated bond types.

Bond type	R_s / Å	R_d / Å	R_{opt} / Å	α
C–C	1.467	1.349	1.388	257.70
C–O	1.367	1.217	1.265	157.38
C–P	1.814	1,64	1.698	118.91
**P–O**	**1.660**	**1.510**	**1.560**	**160.00**

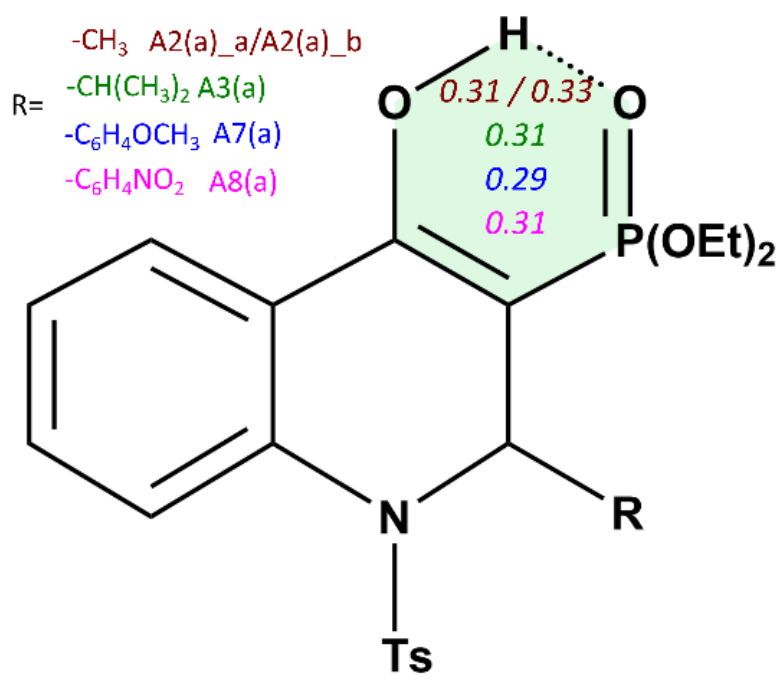

Figure S4. Scheme of the studied *quasi*-ring defined by relevant HOMA indices calculated for structures **A2(a)**, **A3(a)**, **A7(a)**, and **A8(a)**. Two values for **A2(a)_a** and **A2(a)_b** refer to symmetry-independent molecules.

S4. NBO Calculations

Natural Bond Orbital (NBO) analysis[10] was performed using the NBO 6 program[11] as implemented in Gaussian 09 (*RevE.01*).[12]All calculations were carried out at the B3LYP/6-311++G(d,p) level of theory.[13] The molecular geometries were taken directly from single-crystal X-ray diffraction (SCXRD) measurements and used without further geometry optimization or symmetry constraints.
Second-order perturbation stabilization energies, *E*(2), were analyzed to quantify donor–acceptor interactions between occupied (donor) and unoccupied (acceptor) NBOs, with particular focus on hyperconjugative and resonance-type interactions. In addition, Wiberg bond indices (WBI)[14] were extracted from the NBO analysis to assess bond orders and charge distribution.

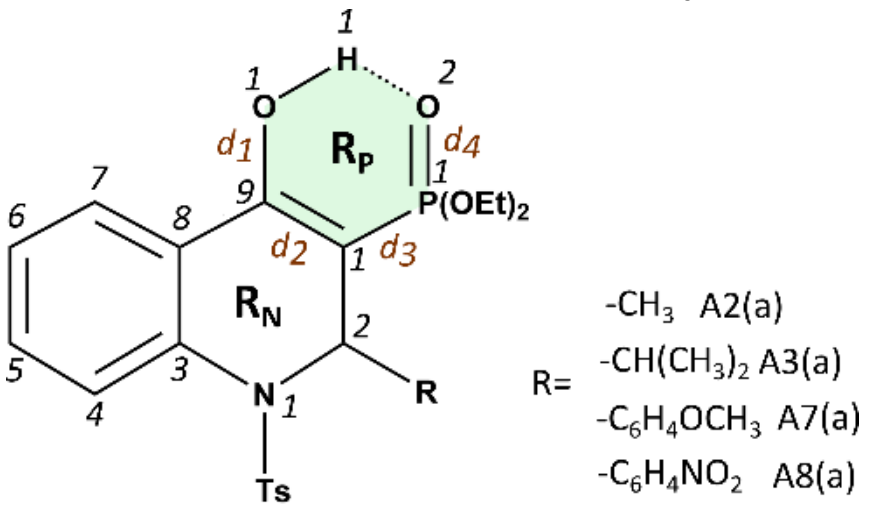

Figure S5. Atom numbering scheme for **A2(a)**, **A3(a)**, **A7(a)**, and **A8(a)**.

Table S15. Selected second-order perturbation energies *E*(2) (kcal mol⁻¹) from NBO analysis at the B3LYP/6-311++G(d,p) level of theory. Only interactions with *E*(2) > 1 kcal mol⁻¹ are listed.

Donor→Acceptor interaction	A2(a)_a	A2(a)_b	A3(a)	A7(a)	A8(a)
n_1(O2)→σ* (O1–H1)	4.15	3.94	5.33	5.61	4.61
n_2(O2)→σ*(O1–H1)	7.09	2.18	1.14	9.28	2.20
n_3 (O2)→σ*(O1–H1)	12.83	17.55	23.22	16.18	20.83
n_1(O2)→σ*(P1–C1)	1.11	1.13	1.20	1.22	1.13
n_2(O2)→σ*(P1–C1)	7.75	3.19	1.97	4.95	3.55
n_3(O2)→σ*(P1–C1)	6.75	10.95	11.85	8.98	10.69
n_2(O2)→σ*(P1–O3)	23.64	25.04	24.20	2.97	24.91
n_3(O2)→σ*(P1–O3)	1.36	-	1.03	21.94	-
n_2(O2)→σ*(P1–O4)	0.70	5.64	8.03	24.84	5.37
n_3(O2)→σ*(P1–O4)	24.55	19.35	16.54	-	19.59
n_1(O2)→RY_6(P1)	5.97	5.78	5.78	5.68	6.00
n_2(O2)→RY_1(P1)	5.07	6.54	7.38	4.61	7.10
n_3(O2)→RY_2(P1)	4.81	5.94	6.71	3.39	6.65
n_1(O3)→σ*(P1–O2)	2.30	3.75	1.56	2.78	1.83
n_2(O3)→σ*(P1–O2)	9.08	6.28	10.23	8.52	10.10
n_1(O3) →σ*(P1–O4)	1.30	-	2.05	1.05	1.84
n_2(O3)→σ*(P1–O4)	8.85	11.45	7.62	9.38	8.39
n_1(O3)→σ*(P1–C1)	2.26	2.31	2.36	2.45	2.36
n_1(O3)→RY_5 (P1)	3.41	3.31	3.48	3.72	3.44
n_1(O4)→σ*(P1–O2)	4.82	5.38	3.50	2.73	5.20

n_2(O4)→σ*(P1–O2)	3.77	3.00	5.76	7.05	3.29
n_1(O4)→σ*(P1–O3)	4.93	4.36	5.06	4.86	4.83
n_2(O4)→σ*(P1–O3)	3.08	3.81	1.32	0.53	3.46
n_1(O4)→RY_3 (P1)	2.10	2.45	1.53	1.36	2.08
n_2(O4)→RY_4(P1)	4.07	4.22	3.56	2.82	4.38
n_1(O1)→σ*(C1–C9)	6.17	6.11	6.29	6.34	6.00
n_2(O1)→π*(C1–C9)	42.86	42.65	43.84	43.88	44.42
σ(P1–O2)→σ*(P1–C1)	1.38	1.30	1.48	1.52	1.45
σ(P1–O2)→σ*(P1–O3)	1.20	1.46	1.16	3.62	1.16
σ(P1–O2)→σ*(P1–O4)	1.98	2.03	1.95	2.02	2.02
σ(P1–O3)→σ*(P1- C1)	1.97	2.08	1.95	2.01	2.05
σ(P1–O3)→σ*(P1–O4)	2.04	2.01	2.18	2.33	1.93
σ(P1–O4) →σ*(P1–C1)	1.69	1.82	1.71	1.73	1.77
σ(P1–O4)→σ*(P1–O2)	1.27	1.24	1.24	1.27	1.25
σ(P1–O4) →σ*(P1–O3)	3.79	3.44	3.84	3.62	3.78
σ(P1–C1)→σ*(P1-O2)	1.14	1.14	1.26	1.24	1.15
σ(P1–C1)→σ*(P1-O3)	1.16	1.12	1.00	0.95	1.14
σ(P1–C1)→σ*(P1-O4)	1.42	1.41	1.53	1.63	1.29
σ(O1–H1)→σ*(C8–C9)	4.97	5.13	4.85	4.91	5.17
σ(O1–C9)→σ*(C1–C9)	1.26	1.27	1.24	1.27	1.24
σ(C1–C2)→σ*(O1–C9)	5.79	5.76	5.47	5.42	5.42
π(C1–C9)→σ*(P1–O3)	5.31	5.12	5.20	4.29	5.37
π(C1–C9)→σ* (P1–O4)	3.69	3.76	4.07	4.81	3.41
π(C1–C9)→σ*(C3–C8)	9.70	9.76	9.37	9.22	8.66
σ(C2–C21)→σ*(C1–C2)	0.69	0.70	0.87	1.18	1.02
σ(C8–C9)→σ*(P1–C1)	3.12	3.13	3.04	3.06	3.07
π(C3-C8)→π*(C1-C9)	16.65	16.26	16.70	16.96	17.79
σ(C8–C9)→σ*(O1–H1)	1.75	1.72	1.70	1.69	1.61
n_1(N1)→σ*(C3–C8)	5.30	5.16	5.12	4.86	5.91
n_2(N1)→π*(C3–C8)	7.92	8.44	9.91	10.13	15.21
n_3(N1)→σ*(C1–C2)	5.64	5.60	4.93	4.85	3.57

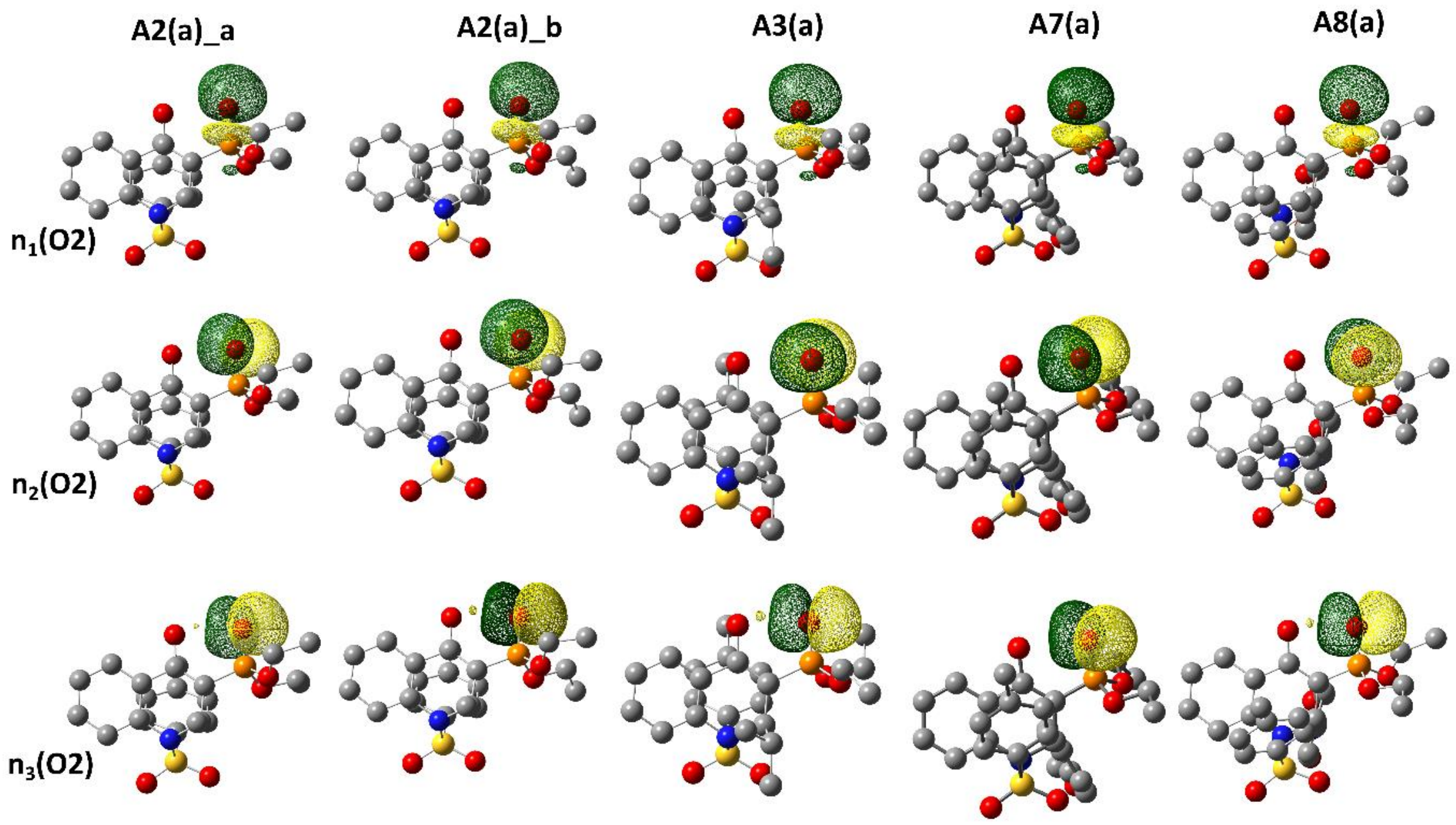

Figure S6. NBO orbital visualization for the O2 lone pairs orbitals (isovalue = 0.05 a.u.)

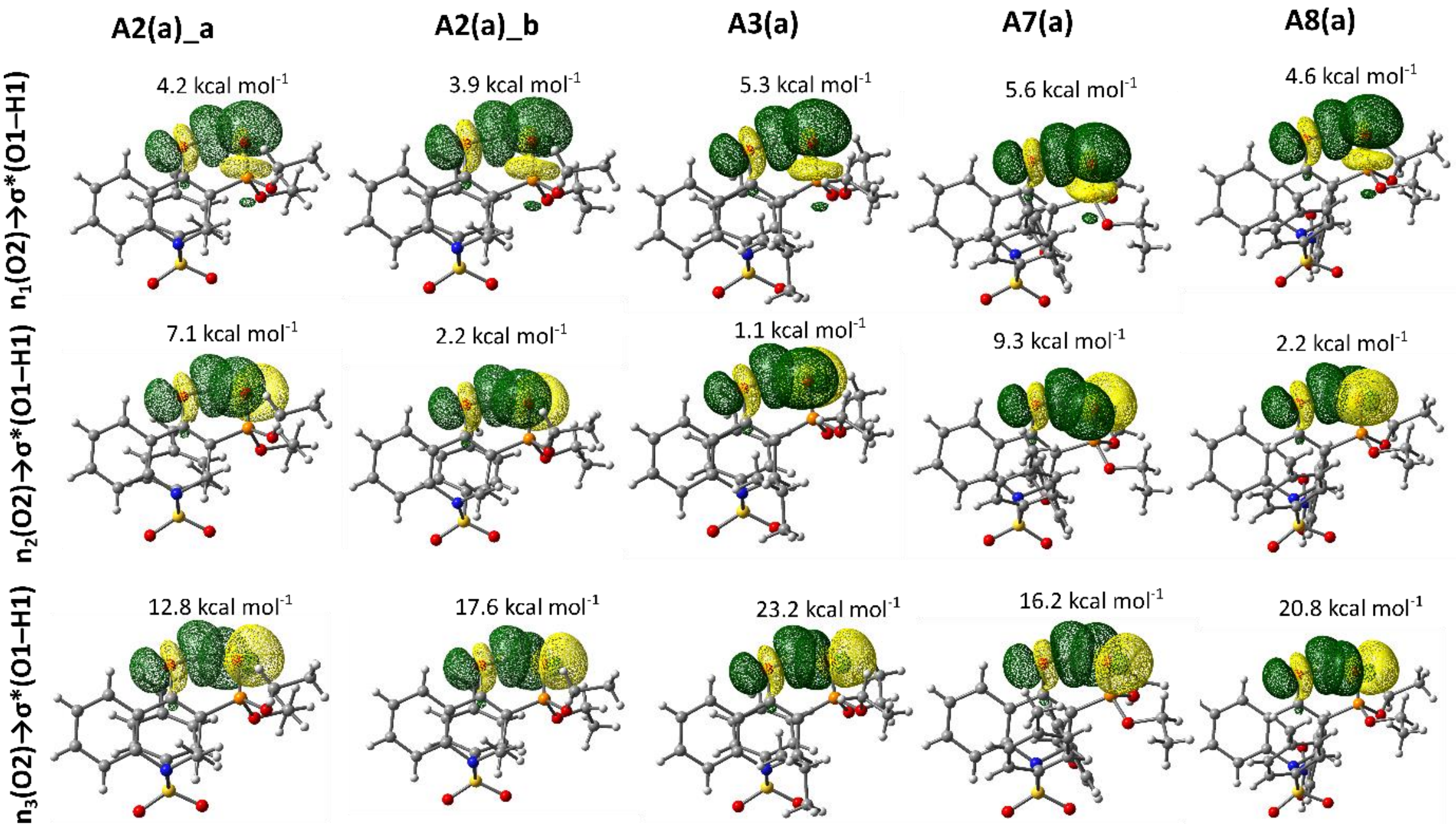

Figure S7. NBO orbital visualization of negative hyperconjugation n(O2)→σ*(O1-H1) defining the intraHB. (isovalue = 0.05 a.u.).

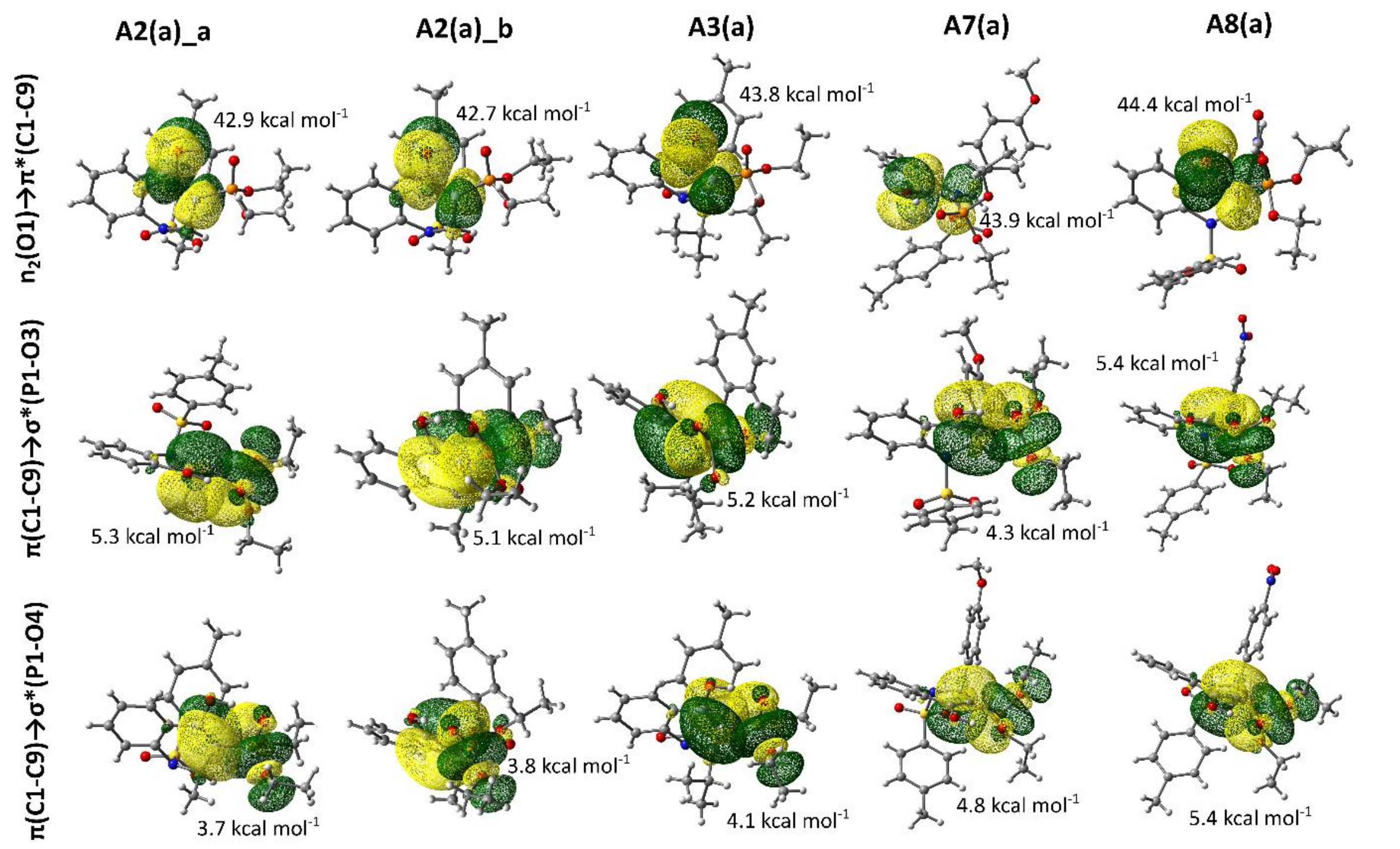

Figure S8. NBO orbital visualization of conjugation n(O1)→π*(C1-C9) and negative hyperconjugation π(C1-C9)→σ*(P1-O3/O4) within quasi-ring R_P (isovalue = 0.05 a.u.).

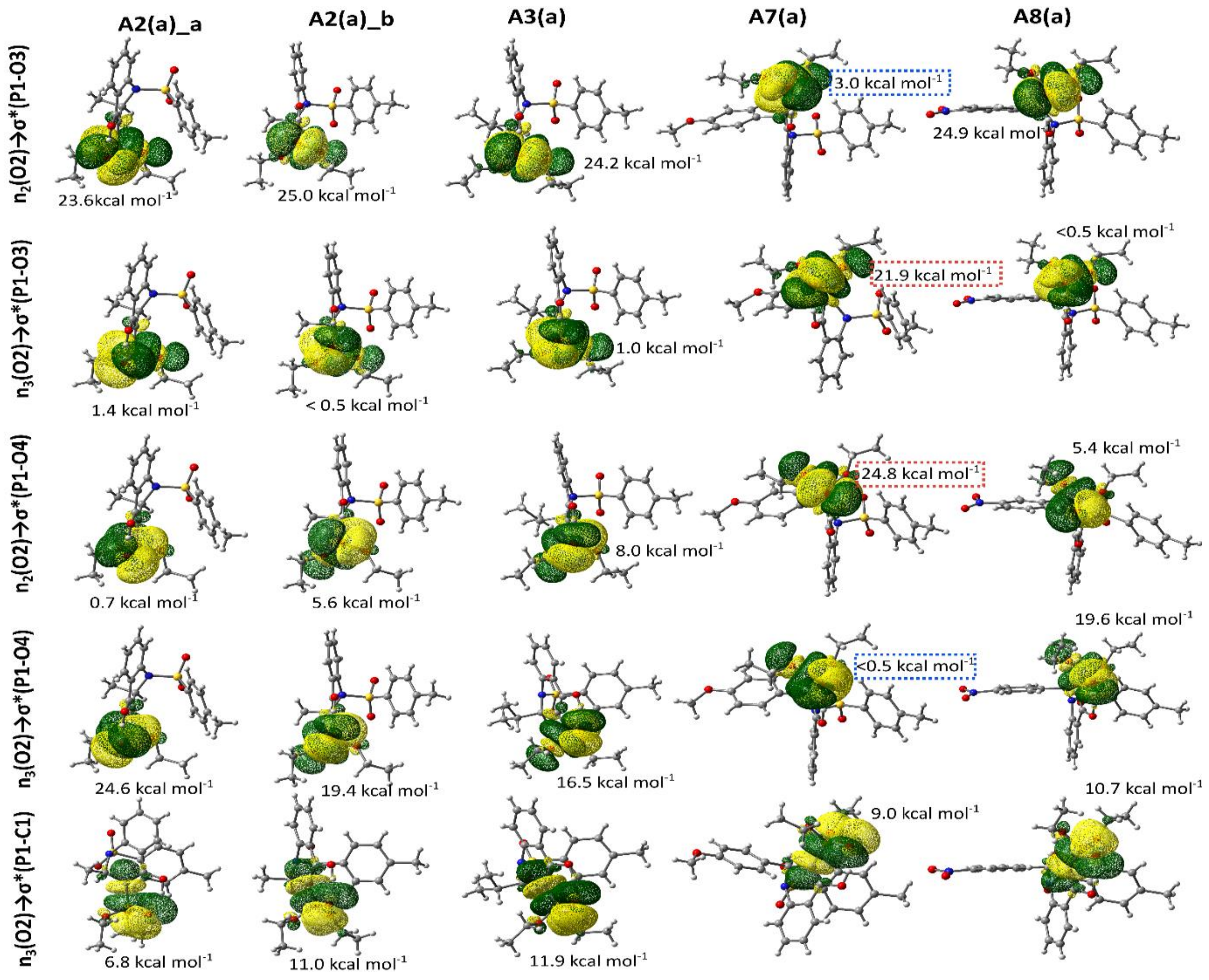

Figure S9. NBO orbital visualization of negative hyperconjugation n(O2)→σ*(P1-O3/O4) (isovalue = 0.05 a.u.).

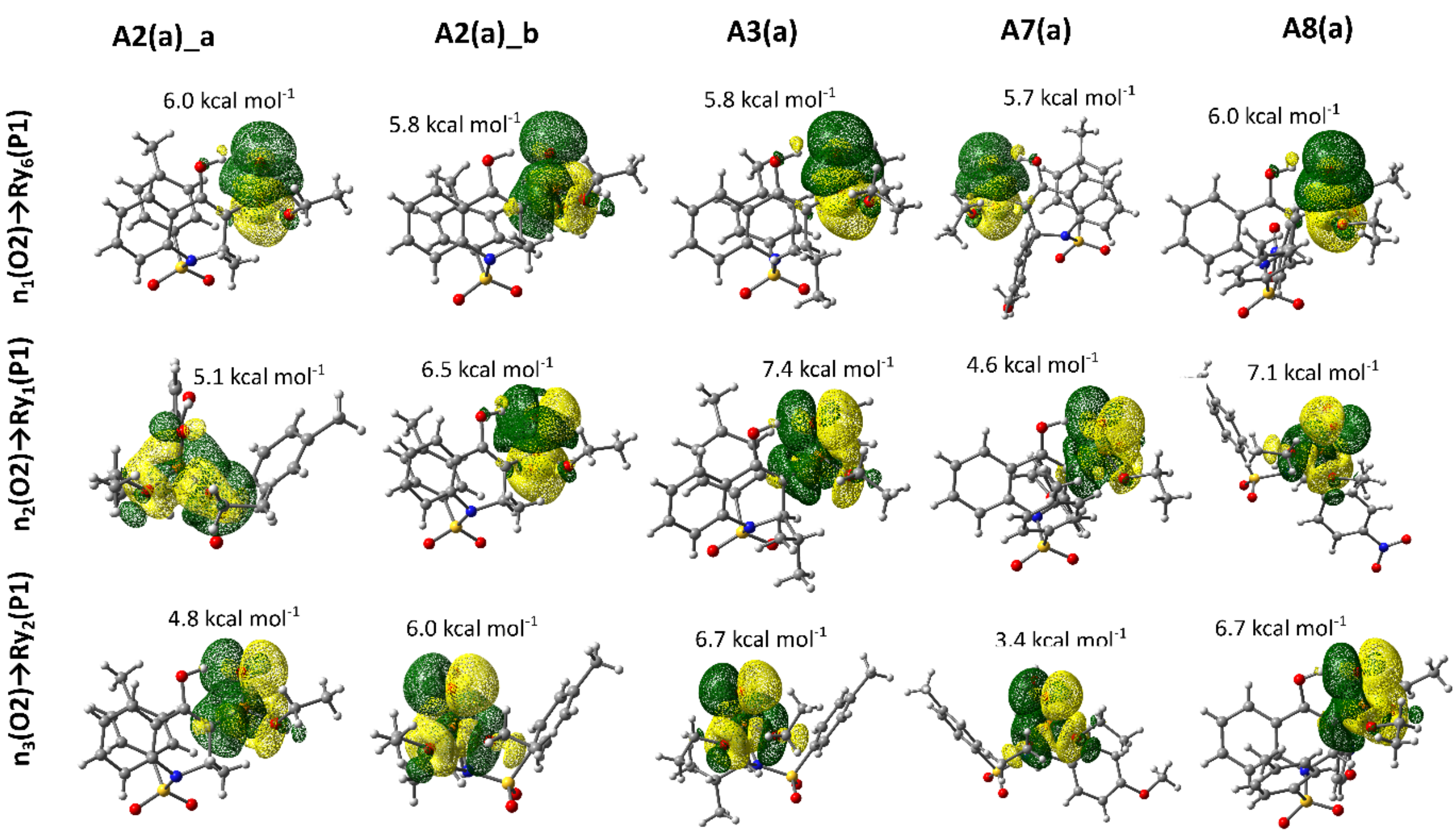

Figure S10. NBO orbital visualization of n(O2) → Rydberg(P) delocalization (isovalue = 0.05 a.u.).

S5. NMR Analysis

a) General comments

^{1}H and $^{31}P\{^{1}H\}$ spectra in CD3CN were recorded on a Bruker AVANCE Neo 400 spectrometer operating at 400 MHz for ^{1}H and 162 MHz for ^{31}P. ^{1}H spectra in C_2D_6SO and in $CDCl_3$ were recorded on a Bruker AVANCE III 500 NMR spectrometer operating at 500 MHz for ^{1}H. For solvent signal presaturation WATERGATE pulse sequence was used.

b) ^{1}H and ^{31}P NMR results

^{1}H and ^{31}P NMR spectra in CD_3CN were registered for **A2(a)**, **A3(a)**, **A7(a)**, **A8(a)**, (Figures S11-S14). In all of these spectra, a singlet signal around 11 ppm is visible at 10.84 ppm, 11.05 ppm, 11.10 ppm, and 11.14 ppm in CD_3CN, respectively. This signal originates from the proton engaged in an intraHB, which results in strong deshielding of this nucleus — a characteristic effect of hydrogen bonding. The NMR spectra show that intramolecular hydrogen bonding occurs in the liquid phase, as in the solid state. For a proton involved in an intramolecular hydrogen bond, high chemical shifts are observed for different kinds of donors and acceptors. However, the particular values of the ^{1}H chemical shift depend on the systems at hand and may be associated with the electronegativity of the atoms in the interacting groups. For example, in the three Schiff bases obtained by condensation of 4-methoxy-, 5-methoxy- and 4,6 dimethoxysalicylaldehyde with methylamine, where the oxygen atom is a donor, and the nitrogen atom is an acceptor, chemical shifts of intramolecularly hydrogen-bonded proton range from 12.83 ppm to 14.04 ppm in $CDCl_3$.[15] When the 4,5-dihydroxynaphthalene-2,7-disulfonate is considered, the Ar-OH oxygen atom is a donor, and the S=O group is an acceptor, and a 17.7 ppm chemical shift in D_2O is registered.[16] Finally, as there are no literature examples of an intramolecular hydrogen bond involving a P=O group as an acceptor, we refer to the values of intermolecular hydrogen bonds of the Ar-OH···O=P type observed for protein-DNA complexes, which were 9.67 and 9.94 ppm.[17] In the studied molecules, the ^{1}H chemical shifts of the Ar-OH···O=P proton are notably higher than the two reported values, indicating a stronger interaction.

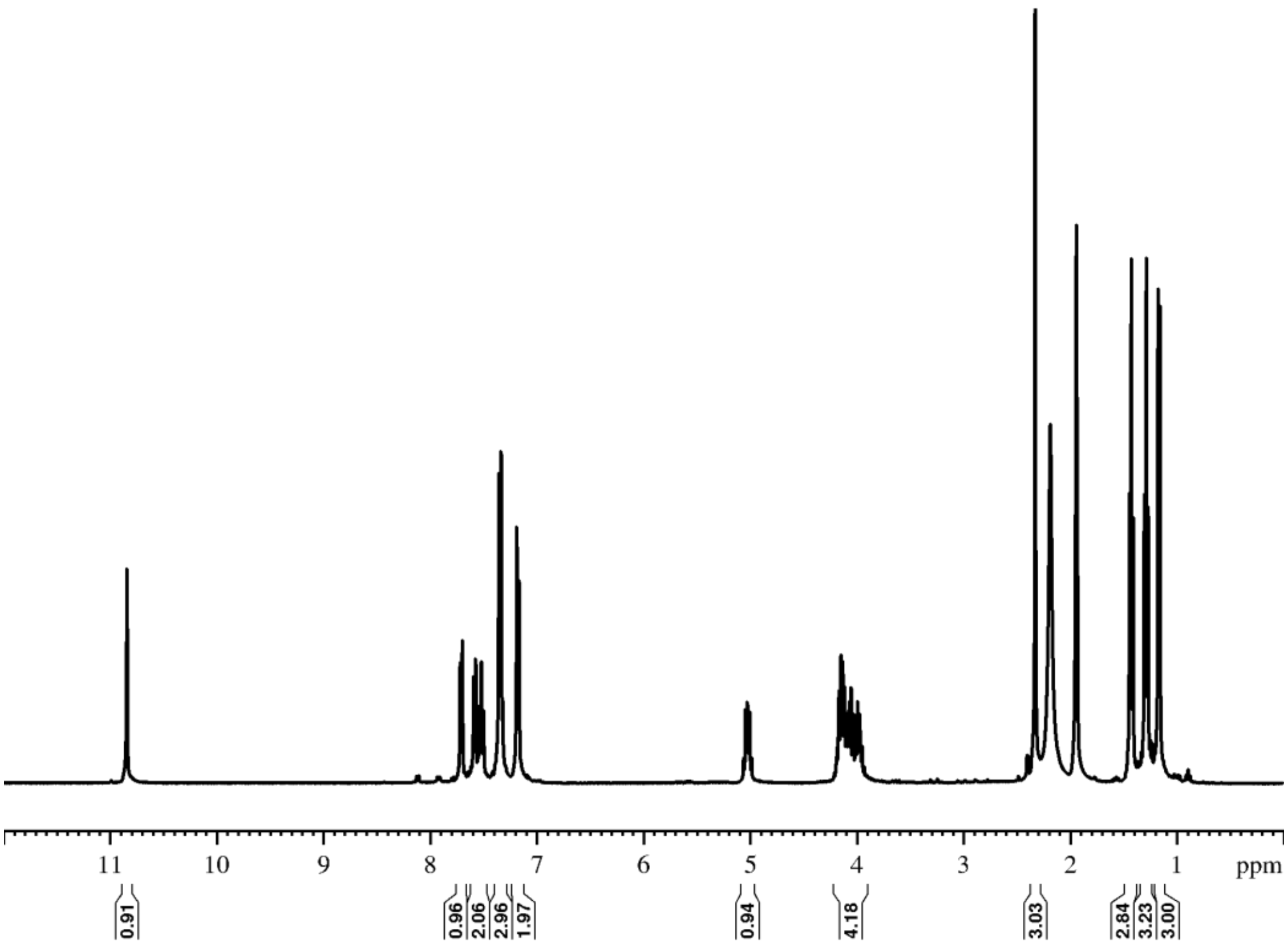

Figure S11. ^{1}H NMR spectrum of **A2(a)** in CD_3CN.

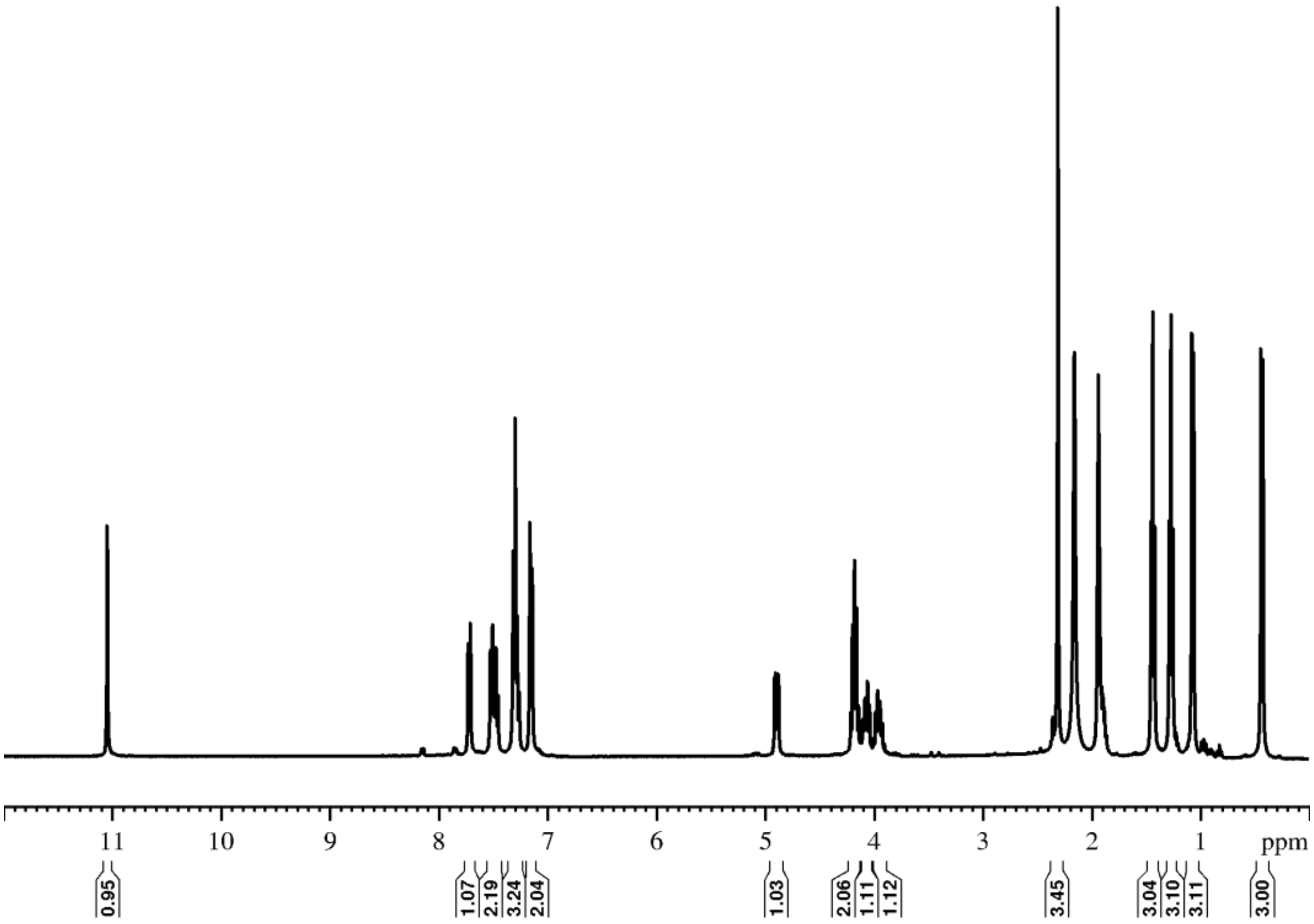

Figure S12. ^{1}H NMR spectrum of **A3(a)** in CD_3CN.

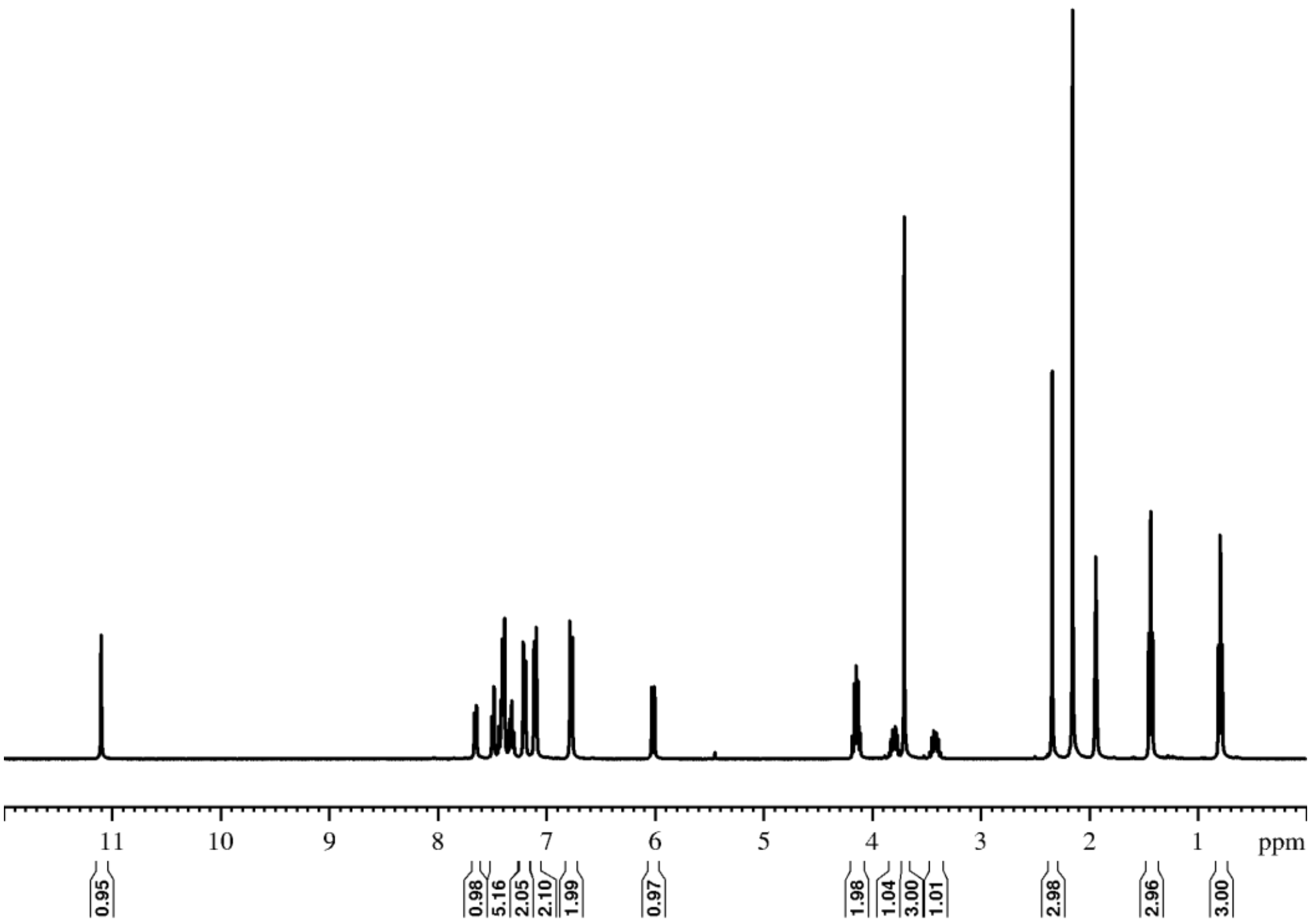

Figure S13. ^{1}H NMR spectrum of **A7(a)** in CD_3CN.

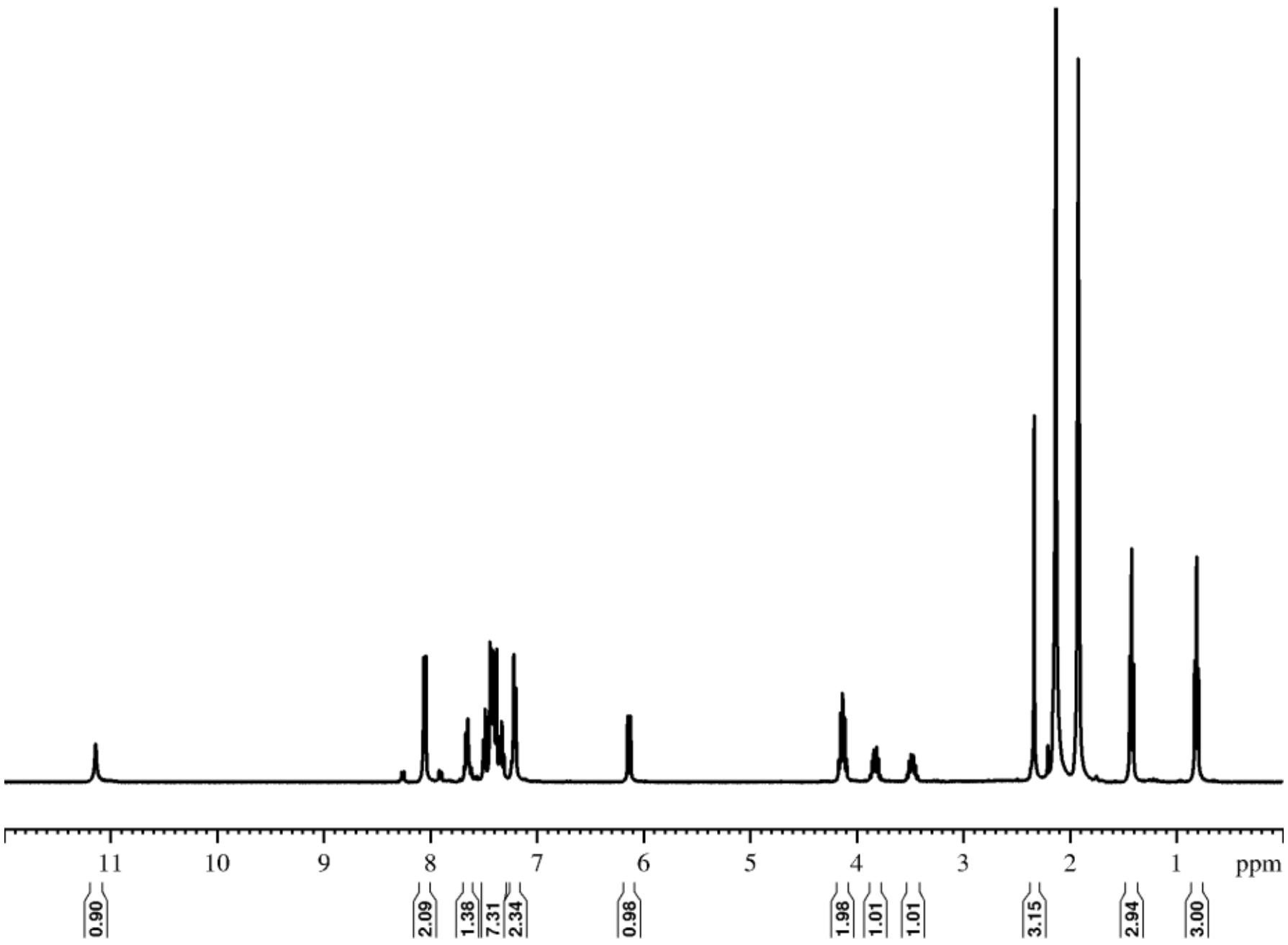

Figure S14. ^{1}H NMR spectrum of **A8(a)** in CD_3CN.

In the ^{1}H and $^{31}P\{^{1}H\}$ NMR spectra (Figures S14-S15), the effect of the substituent at the C2 atom on the chemical shifts (and hence, on the electron density) of the ^{1}H and ^{31}P nuclei can be observed. ^{31}P NMR spectra were registered with proton decoupling. For the **A7(a)**, **A2(a)**, **A3(a)** with electron donating group (EDG) at C2 position, the chemical shifts of phosphorus increased with the changing substituent from isopropyl, methyl to metoxyphenyl. In **A8(a),** the phosphorus chemical shift is the lowest of all the investigated compounds, and the substituent at the C2 position is an electron-withdrawing (EWG) nitro-group. Based on the ^{31}P chemical shift differences, it may be assumed that the most substantial shielding effect is present in the molecule with EWG substituent. At the ^{1}H spectra, the chemical shift of the signal around 11 ppm, originating from the proton engaged in an intramolecular hydrogen bond with the phosphoryl group, increased with the size of the substituent, and was the highest for **A8(a)** with an electron-withdrawing (EWG) nitrophenyl-group. Also, the deshielding effect increased in the same manner as the chemical shift for this particular proton. For the most deshielded proton, the chemical shift is 11.14 ppm, and for the most shielded, it is 10.84 ppm. However, the noted differences are not sufficiently large to conclude that the substituent effect is pivotal for the strength of the intramolecular HB in the analyzed systems.

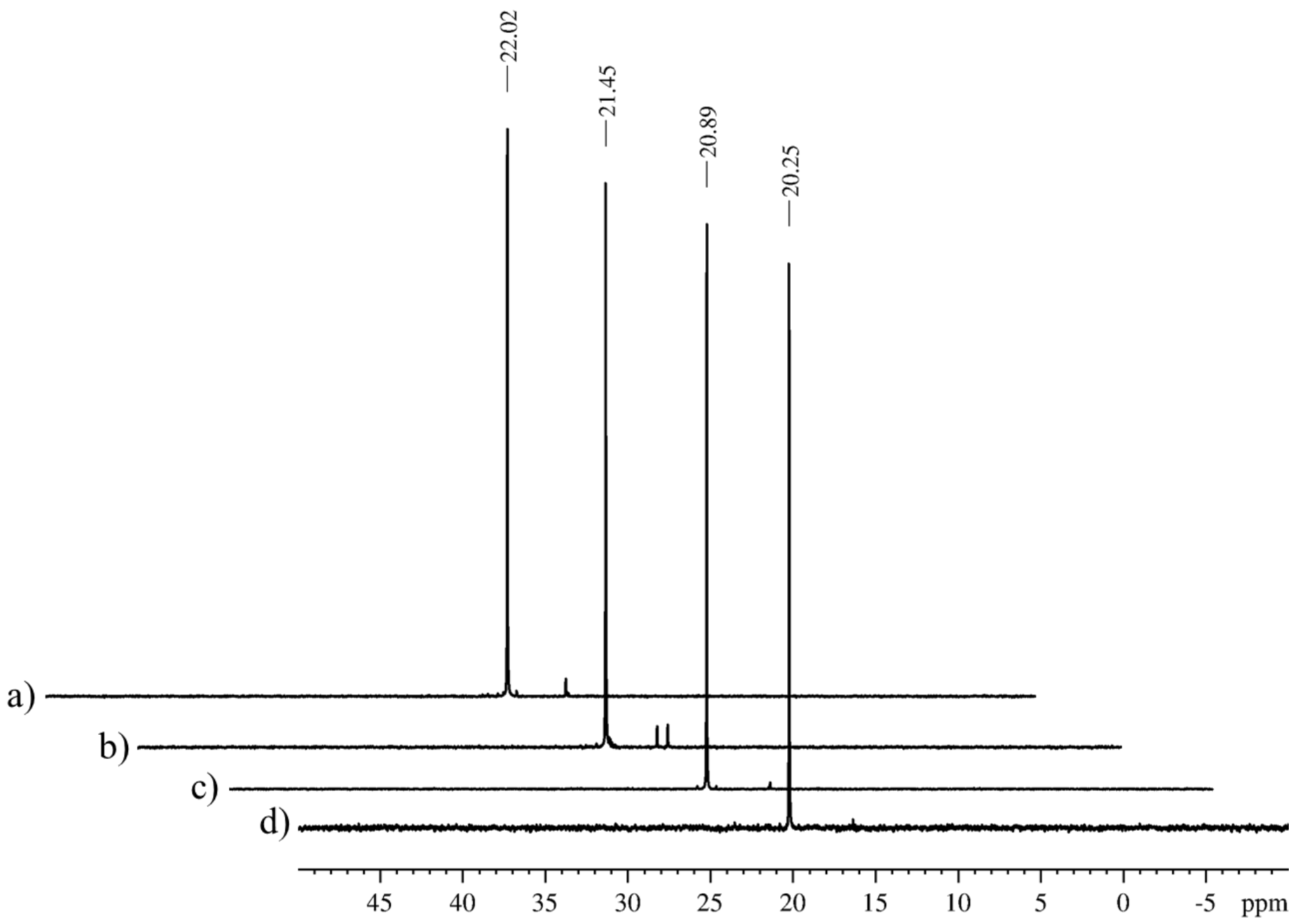

Figure S15. $^{31}P\{^{1}H\}$ NMR spectrum of a) **A3(a)**, b) **A2(a)**, c) **A7(a)**, d) **A8(a)** in CD_3CN.

c) Concentration-dependent 1H NMR spectroscopy

To evaluate the strength of the intramolecular hydrogen bonds in the investigated compounds in solution, ^{1}H NMR spectra were recorded at different concentrations in deuterated dimethyl sulfoxide (C_2D_6SO), a strongly electron-donating solvent known to break intramolecular hydrogen bonds.[18] For the measurements, several low concentrations of each compound in C_2D_6SO were prepared. The pulse program for the most diluted samples was modified because compound signals were absent in some cases; therefore, residual dimethyl sulfoxide and water signals were suppressed. The signal from the proton engaged in an intramolecular hydrogen bond around 11 ppm was integrated (Table S16, Figure S17) relative to the signal at 1.36-1.37 ppm (originating from the methyl group), which was set to 3.0.

Table S16. Summary of ^{1}H NMR spectra analysis in C_2D_6SO for different concentrations.

**A2(a)**		**A3(a)**		**A7(a)**		**A8(a)**		Pulse program
^{1}H integration ppm	Concentration mmol/dm^{3}	^{1}H integration ppm	Concentration mmol/dm^{3}	^{1}H integration ppm	Concentration mmol/dm^{3}	^{1}H integration ppm	Concentration mmol/dm^{3}	
0.96	2.44	0.92	1.95	0.95	2.21	0.96	1.89	Standard ^{1}H program
0.79	0.97	0.86	0.78	1.01	0.88	0.91	0.75	Standard ^{1}H program
0.83	0.49	0.75	0.39	0.78	0.44	0.98	0.38	Standard ^{1}H program
0.86	0.24	0.85	0.2	0.78	0.22	0.67	0.19	With DMSO and water presaturation
0.24	0.12	0.44	0.1	No signal	0.11	No signal	0.09	With DMSO and water presaturation

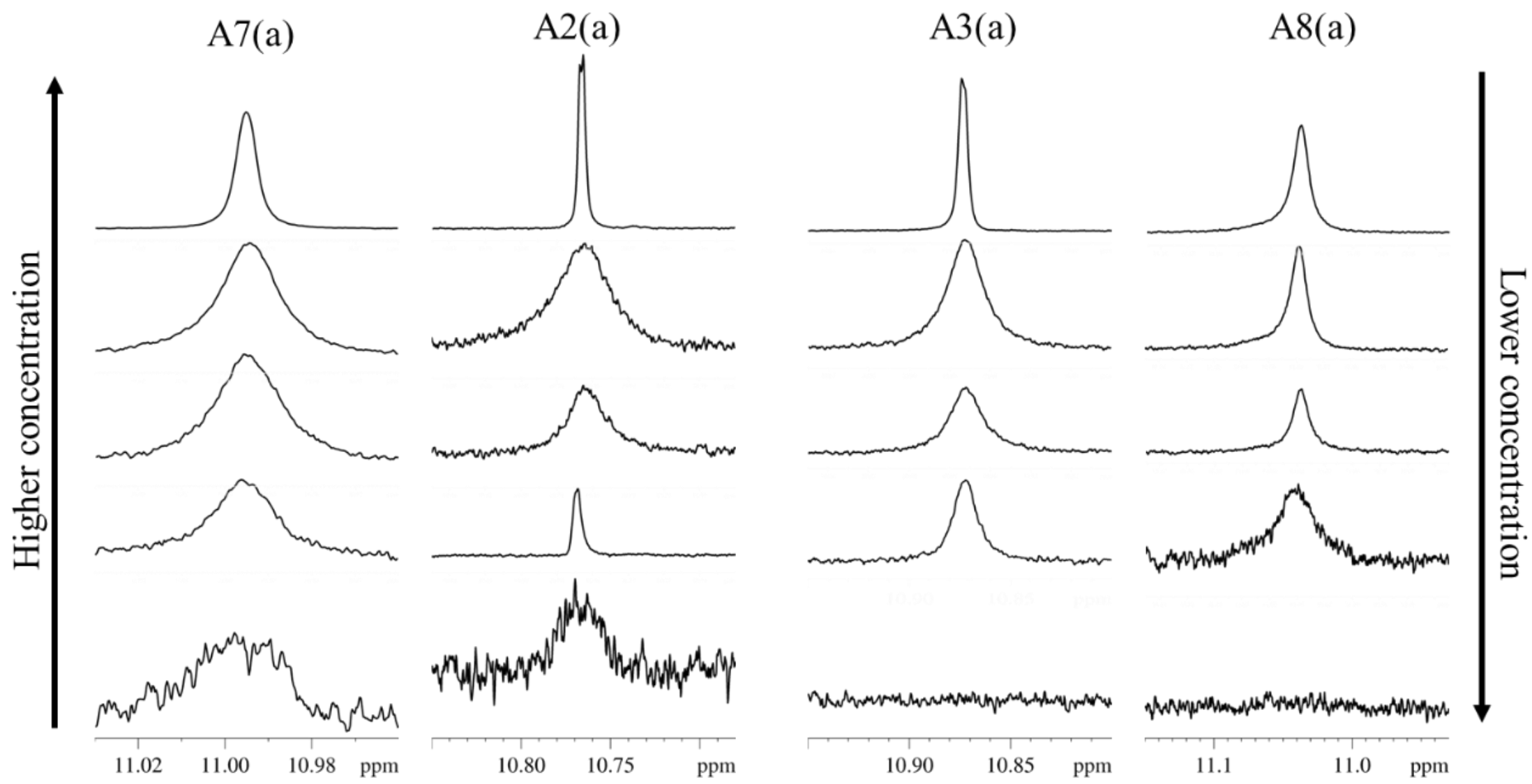

Figure S16. Signals originating from the proton involved in the intramolecular hydrogen bond for different concentrations in C_2D_6SO. Because solvent and water presaturation was applied at the lowest concentrations, the linewidths and relative intensities of these spectra are not intended for quantitative comparison with those obtained using the standard ^{1}H experiment

For the most diluted samples of **A3(a)** and **A8(a),** the signals from the protons involved in the intramolecular hydrogen bond disappeared entirely. In contrast, for **A7(a)** and **A2(a)** the observed signals are very weak. For the remaining concentrations, the OH resonance remains detectable (Table S16), although its linewidth varies non-monotonically. Quantitative comparison of the linewidths and integrals is precluded by the use of presaturation and lower signal-to-noise ratios at the two lowest concentrations. The nearly constant OH chemical shift over the range in which the signal is reliably detectable is consistent with persistence of the intramolecularly hydrogen-bonded motif, although rapid exchange with solvent- or water-associated states cannot be excluded.

Abrahm's (A_{NMR}) parameter determination

Additionally, to describe the strength of the intramolecular hydrogen bonds in the investigated compounds Abrahms's parameter (A_{NMR}) was used. ^{1}H spectra of analyzed compounds in $CDCl_3$ and C_2D_6SO were registered, and based on the chemical shifts originating from the proton involved in the intramolecular hydrogen bond, the parameter A_{NMR} can be calculated[19]:

$$A_{NMR} = 0.0065 + 0.133 \cdot \Delta\delta$$

where: *Δδ* is the difference in ppm between the chemical shift of the proton involved in the intramolecular hydrogen bond in C_2D_6SO and $CDCl_3$.
The chemical shifts and the determined parameters A_{NMR} are summarized in Table S17.

Table S17. ^{1}H chemical shifts of the proton involved in the intramolecular hydrogen bond in C_2D_6SO, $CDCl_3$, and the determined A_{NMR} for **A2(a)**, **A3(a)**, **A7(a)**, **A8(a)**.

	**δ_{C2D6SO} [ppm]**	**δ_{CDCl3} [ppm]**	**Δδ [ppm]**	**A_{NMR}[-]**
**A2(a)**	10.76	10.59	0.17	0.029
**A3(a)**	10.87	10.80	0.07	0.015
**A7(a)**	10.99	10.86	0.13	0.023
**A8(a)**	11.04	10.92	0.12	0.022

Based on the criterion $A_{NMR}<0.1$ for aromatic or aliphatic OH groups described in the work mentioned above, the values indicate the formation of strong intramolecular hydrogen bonds ($A_{NMR}\approx0$)

S6. IR Analysis

Fourier transform infrared spectroscopy (FTIR) measurements were performed on a Thermo Scientific Nicolet 6700 instrument with an attenuated total reflectance (ATR) GoldenGate accessory (Waltham, MA, USA). For solid, 64 scans were registered; for liquid, 128. The intensities in the selected range of the spectra are increased by a factor of 4 for better clarity.

FTIR spectra were recorded in the solid and liquid phases (~20% solution of each compound in CH_2Cl_2) for **A2(a)**, **A3(a)**, **A7(a)**, **A8(a)**. Comparing these spectra revealed information about intramolecular hydrogen bonding (intraHB) in both phases. In FTIR spectra (Figures S17-S20) of solutions of particular compounds, we observe signals at ca. 3000 cm^{-1}. The hydrogen-bonded ν(O–H) mode contributes to the broad underlying absorption envelope extending approximately from 3200 to 2700 cm^{-1}. Because of extensive overlap with ν(C–H) vibrations, the local maxima at 2960–2985 cm^{-1} are not assigned as discrete ν(O–H) frequencies. This broad absorption envelope is red-shifted relative to the ν(O–H) band of a non-hydrogen-bonded hydroxyl group, typically observed at approximately 3700–3450 cm^{-1}, consistent with hydrogen-bond-induced weakening of the O–H bond. The comparable shape and position of the envelope in the solid state and in CH_2Cl_2 solution are consistent with the presence of the intramolecular O–H···O=P motif in both phases.

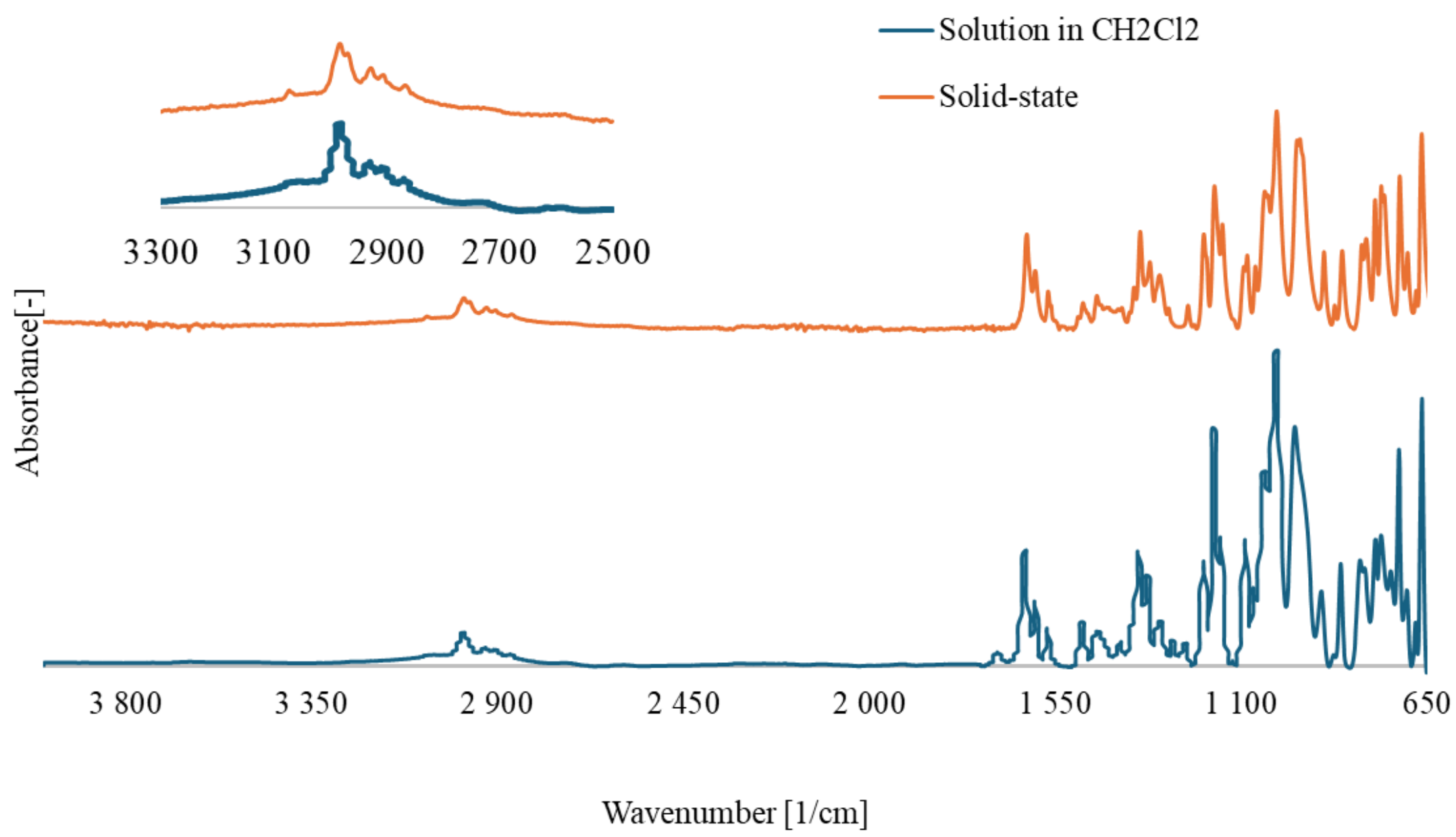

Figure S17. Comparison of FTIR spectra of **A2(a)** in solid-state and in CH_2Cl_2 solution. The broad 3200–2700 cm^{-1} envelope contains overlapping ν(O–H) and ν(C–H) contributions

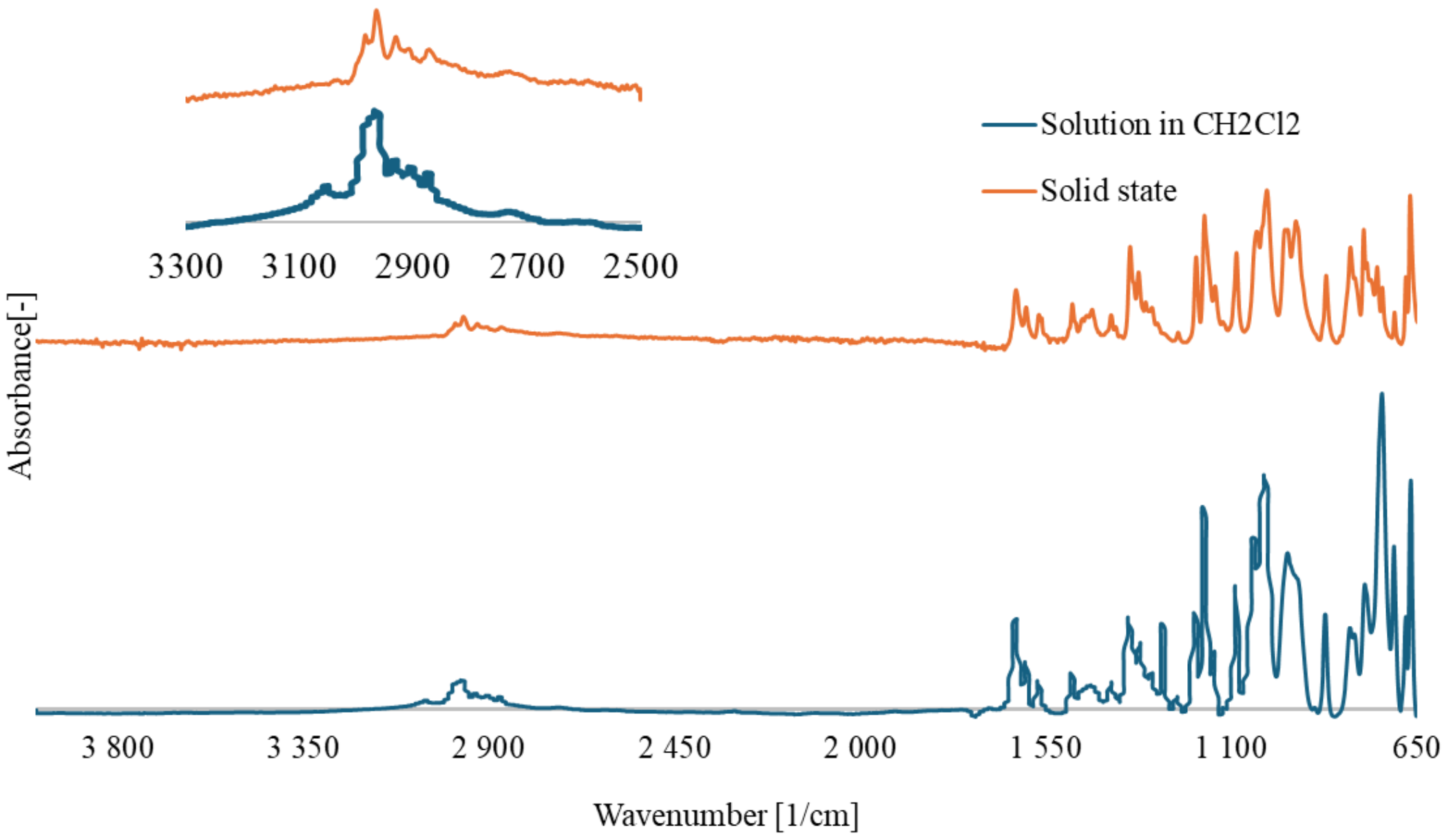

Figure S18. Comparison of FTIR spectra of **A3(a)** in solid-state and in CH_2Cl_2 solution. The broad 3200–2700 cm^{-1} envelope contains overlapping ν(O–H) and ν(C–H) contributions

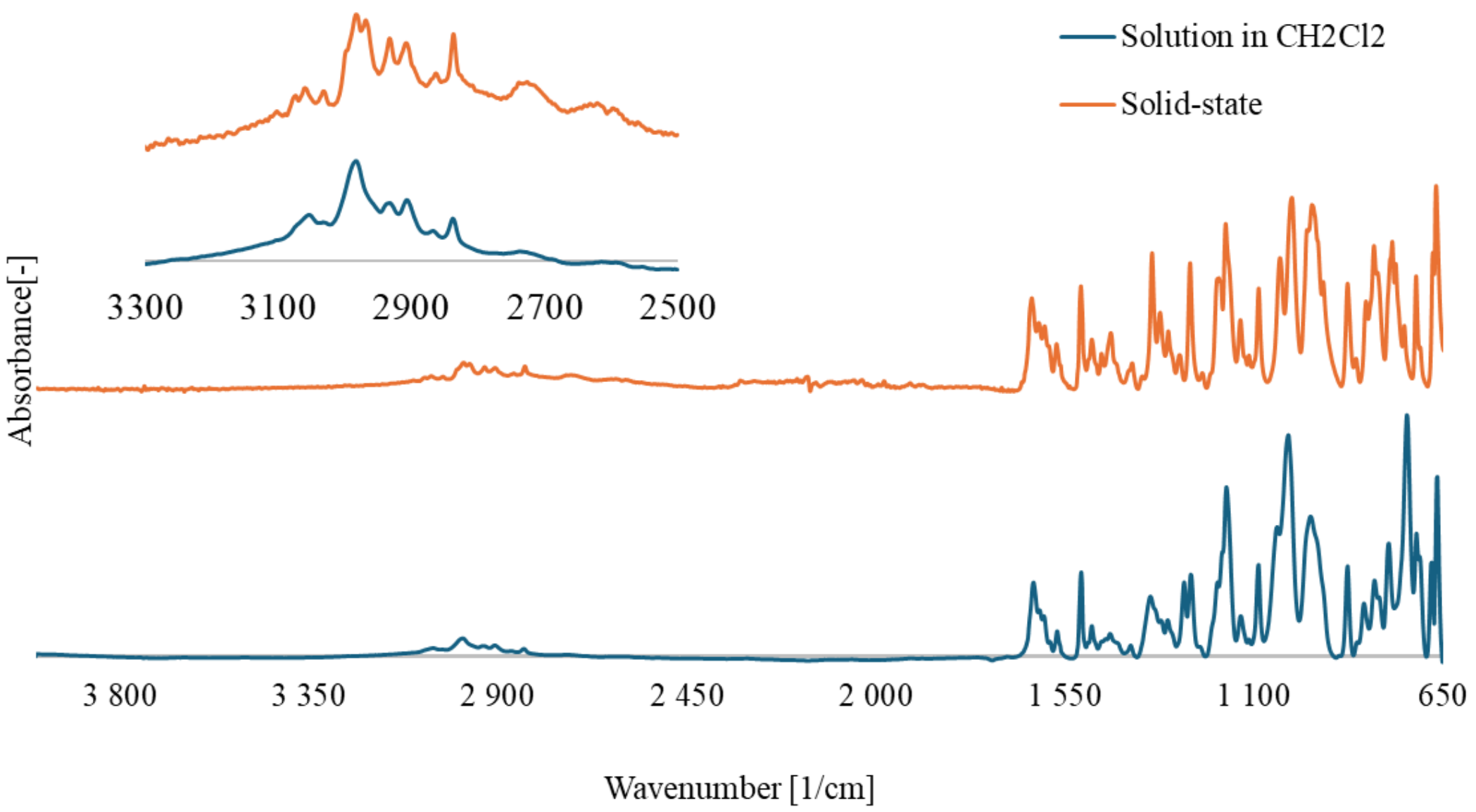

Figure S19. Comparison of FTIR spectra of **A7(a)** in solid-state and in CH_2Cl_2 solution. The broad 3200–2700 cm^{-1} envelope contains overlapping ν(O–H) and ν(C–H) contributions

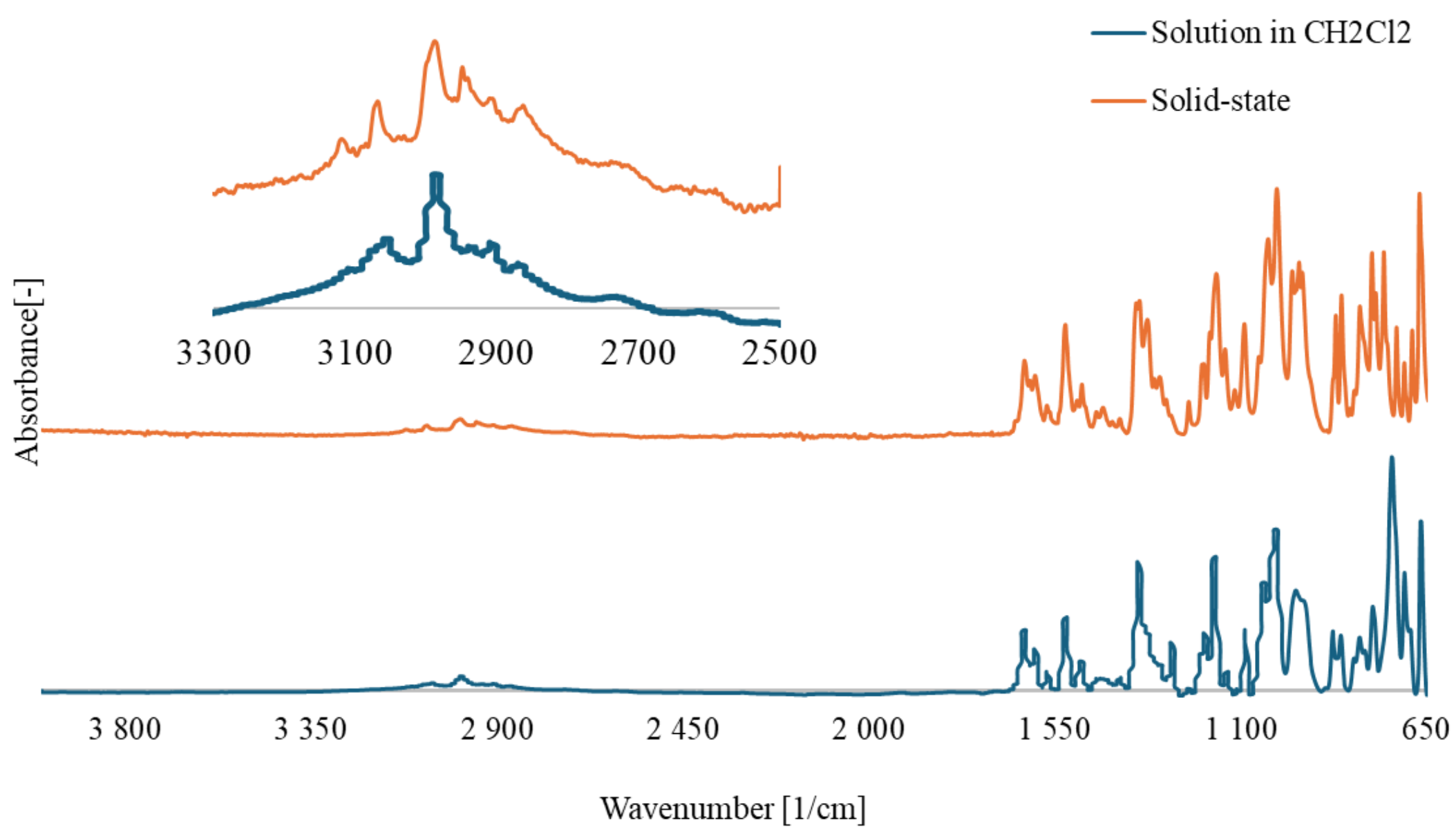

Figure S20. Comparison of FTIR spectra of **A8(a)** in solid-state and in CH2Cl2 solution. The broad 3200–2700 cm^{-1} envelope contains overlapping ν(O–H) and ν(C–H) contributions

References:

[1] Agilent (2014). *CrysAlis PRO*. Agilent Technologies Ltd, Yarnton, Oxfordshire, England.
[2] G. M. Sheldrick, "SHELXT - Integrated space-group and crystal-structure determination" *Acta Crystallographica Section A* **71** (2015): 3–8.
[3] G. M. Sheldrick, "Crystal structure refinement with SHELXL" *Acta Crystallographica Section C* **71** (2015): 3–8.
[4] C. B. Hübschle, G. M. Sheldrick, B. Dittrich, "ShelXle: A Qt graphical user interface for SHELXL" *Journal of Applied Crystallography* **44** (2011): 1281–1284.
[5] D. A. Keen, M. J. Gutmann, C. C. Wilson, "SXD - The single-crystal diffractometer at the ISIS spallation neutron source" *Journal of Applied Crystallography* **39** (2006): 714–722.
[6] M. J. Gutmann, "A 3D profile function suitable for integration of neutron time-of-flight single crystal diffraction peaks" *Nuclear Instruments and Methods in Physics Research A* **848** (2017): 170–173.
[7] T. M. Krygowski, M. K. Cyrański, "Structural aspects of aromaticity" *Chemical Reviews* **101** (2001): 1385–1419.
[8] H. Szatyłowicz, P. A. Wieczorkiewicz, T. M. Krygowski, "Aromaticity Concepts Derived from Experiments" *Sci* **4** (2022): 1–22.
[9] B. Gamoke, D. Neff, J. Simons, "Nature of PO bonds in phosphates" *Journal of Physical Chemistry A* **113** (2009), 5677–5684.
[10] J. P. Foster, F. Weinhold, "Natural Hybrid Orbitals "*Journal of the American Chemical Society*, **102** (1980): 7211-7218.
[11] E. D. Glendening, C. R. Landis, F. Weinhold, "NBO 6.0: Natural bond orbital analysis program" *Journal of Computational Chemistry Comput. Chem.* **2013**, *34*, 1429–1437.
[12] Gaussian 09, Revision E.01, M. J. Frisch, G. W. Trucks, H. B. Schlegel, G. E. Scuseria, M. A. Robb, J. R. Cheeseman, G. Scalmani, V. Barone, G. A. Petersson, H. Nakatsuji, X. Li, M. Caricato, A. Marenich, J. Bloino, B. G. Janesko, R. Gomperts, B. Mennucci, H. P. Hratchian, J. V. Ortiz, A. F. Izmaylov, J. L. Sonnenberg, D. Williams-Young, F. Ding, F. Lipparini, F. Egidi, J. Goings, B. Peng, A. Petrone, T. Henderson, D. Ranasinghe, V. G. Zakrzewski, J. Gao, N. Rega, G. Zheng, W. Liang, M. Hada, M. Ehara, K. Toyota, R. Fukuda, J. Hasegawa, M. Ishida, T. Nakajima, Y. Honda, O. Kitao, H. Nakai, T. Vreven, K. Throssell, J. A. Montgomery, Jr., J. E. Peralta, F. Ogliaro, M. Bearpark, J. J. Heyd, E. Brothers, K. N. Kudin, V. N. Staroverov, T. Keith, R. Kobayashi, J. Normand, K. Raghavachari, A. Rendell, J. C. Burant, S. S. Iyengar, J. Tomasi, M. Cossi, J. M. Millam, M. Klene, C. Adamo, R. Cammi, J. W. Ochterski, R. L. Martin, K. Morokuma, O. Farkas, J. B. Foresman, and D. J. Fox, Gaussian, Inc., Wallingford CT, 2016.
[13] A. D. Becke, "Density-functional thermochemistry. III. The role of exact exchange" *The Journal of Chemical Physics **98*** (1993): 5648–5652.
[14] K. B. Wiberg, "Application of the Pople-Santry-Segal CNDO method to the cyclopropylcarbinyl and cyclobutyl cation and to bicyclobutane" *Tetrahedron* **24** (1968): 1083–1096.
[15] W. Schilf, J. P. Bloxsidge, J. R. Jones, S. Y. Lu, "Investigations of intramolecular hydrogen bonding in three types of Schiff bases by ^{2}H and ^{3}H NMR isotope effects" *Magnetic Resonance in Chemistry* **42** (2004): 556–560.
[16] S. Zhou, L. Wang, "Symmetry and ^{1}H NMR chemical shifts of short hydrogen bonds: Impact of electronic and nuclear quantum effects" *Physical Chemistry Chemical Physics* **22** (2020): 4884–4895.
[17] B. Yu, C. C. Pletka, J. Iwahara, "NMR Observation of Intermolecular Hydrogen Bonds between Protein Tyrosine Side-Chain OH and DNA Phosphate Groups" *Journal of Physical Chemistry B* **124** (2020): 1065–1070.
[18] M. K. D. Jamróz, S. Kaźmierski, K. Stefaniak, V. B. Gliński, J. A. Gliński, "Conformational equilibria in selected A-type trimeric procyanidins" *Organic Biomolecular Chemistry* **12** (2014): 9837–9844.

[19] M. H. Abraham, R. J. Abraham, W. E. Acree, A. E. Aliev, A. J. Leo, W. L. Whaley, "An NMR method for the quantitative assessment of intramolecular hydrogen bonding; Application to physicochemical, environmental, and biochemical properties" *Journal of Organic Chemistry* **79** (2014): 11075–11083.
